# Reduced circulating polyamines in polyendocrine metabolic ovarian syndrome patients and the impact of putrescine on ovarian function and fertility in a murine PMOS model^†^

**DOI:** 10.1093/biolre/ioag139

**Published:** 2026-07-02

**Authors:** Subrata Das, Joseph R D Fernandes, P T Nikhil, S Narayana, Gayatri Shinde, Srabani Mukherjee, Chandrika Anand, N Suchetha Kumari, Sridev Mohapatra, Arnab Banerjee

**Affiliations:** Department of Biological Sciences, Birla Institute of Technology and Science Pilani K K Birla Goa Campus, Zuarinagar, Sancoale, Goa, India; Department of Biological Sciences, Birla Institute of Technology and Science Pilani K K Birla Goa Campus, Zuarinagar, Sancoale, Goa, India; Department of Biological Sciences, Birla Institute of Technology and Science-Pilani, Hyderabad, Telangana, India; Department of Biochemistry, Padmashree Institute of MLT, Bangalore, Karnataka, India; Department of Molecular Endocrinology, ICMR National Institute for Research on Women's Health, Mumbai, Maharashtra, India; Department of Molecular Endocrinology, ICMR National Institute for Research on Women's Health, Mumbai, Maharashtra, India; Department of Gynaecology and Obstetrics, Saraswathi Speciality Clinic, Bangalore, Karnataka, India; Department of Biochemistry, Shrinivas University, Mangalore, Karnataka, India; Department of Biological Sciences, Birla Institute of Technology and Science-Pilani, Hyderabad, Telangana, India; Department of Biological Sciences, Birla Institute of Technology and Science Pilani K K Birla Goa Campus, Zuarinagar, Sancoale, Goa, India; Department of Zoology, Banaras Hindu University, Varanasi, Uttar Pradesh, India

**Keywords:** PMOS, polyamines, putrescine, GnRH, ovary, uterus

## Abstract

GnRH is essential for regulating the reproductive system in mammals. Recent research has shown that polyamines, biogenic amines produced in body, can regulate gonadotropin-releasing hormone (GnRH) levels. These polyamine levels are affected by metabolic disorders like diabetes and obesity. However, it remains unclear whether changes in polyamine expression are related to polyendocrine metabolic ovarian syndrome (PMOS), which is characterized by altered GnRH patterns. Circulatory polyamine levels were measured in PMOS patients. In a study on prepubertal female Balb/c mice induced with a PMOS-like condition using DHEA, the mice were treated with polyamines (putrescine, agmatine, and DFMO) for 5 days. The effects on body weight, serum glucose, testosterone, GnRH, GnIH levels, and gene expression related to ovarian inflammation and folliculogenesis were observed. The study found that circulatory polyamines (putrescine, spermidine, and spermine) are lower in PMOS patients than in healthy individuals. A single dose of putrescine improved PMOS-like symptoms in mice by reducing GnRH, glucose, testosterone levels, body weight, and inflammation in the ovary. Exogenous polyamine treatment increased endogenous polyamine markers in the hypothalamus and ovary, thereby improving fertility and pregnancy outcomes in PMOS mice by regulating key uterine markers. These findings suggest that circulating polyamine levels vary between PMOS and healthy individuals. Exogenous polyamine treatment enhances GnRH metabolism in the hypothalamus by modulating endogenous polyamine genes, thereby improving PMOS traits and ovarian morphology, reducing serum testosterone levels, and supporting successful pregnancy. The authors thereby suggest that endogenous polyamines are key to the pathophysiology of PMOS, and that exogenous polyamines could be a potential target for PMOS management.

## Teaser

Exogenous polyamine administration ameliorates the disrupted reproductive and metabolic parameters associated with PMOS in murine models.

## Graphical Abstract

**Graphical Abstract:**
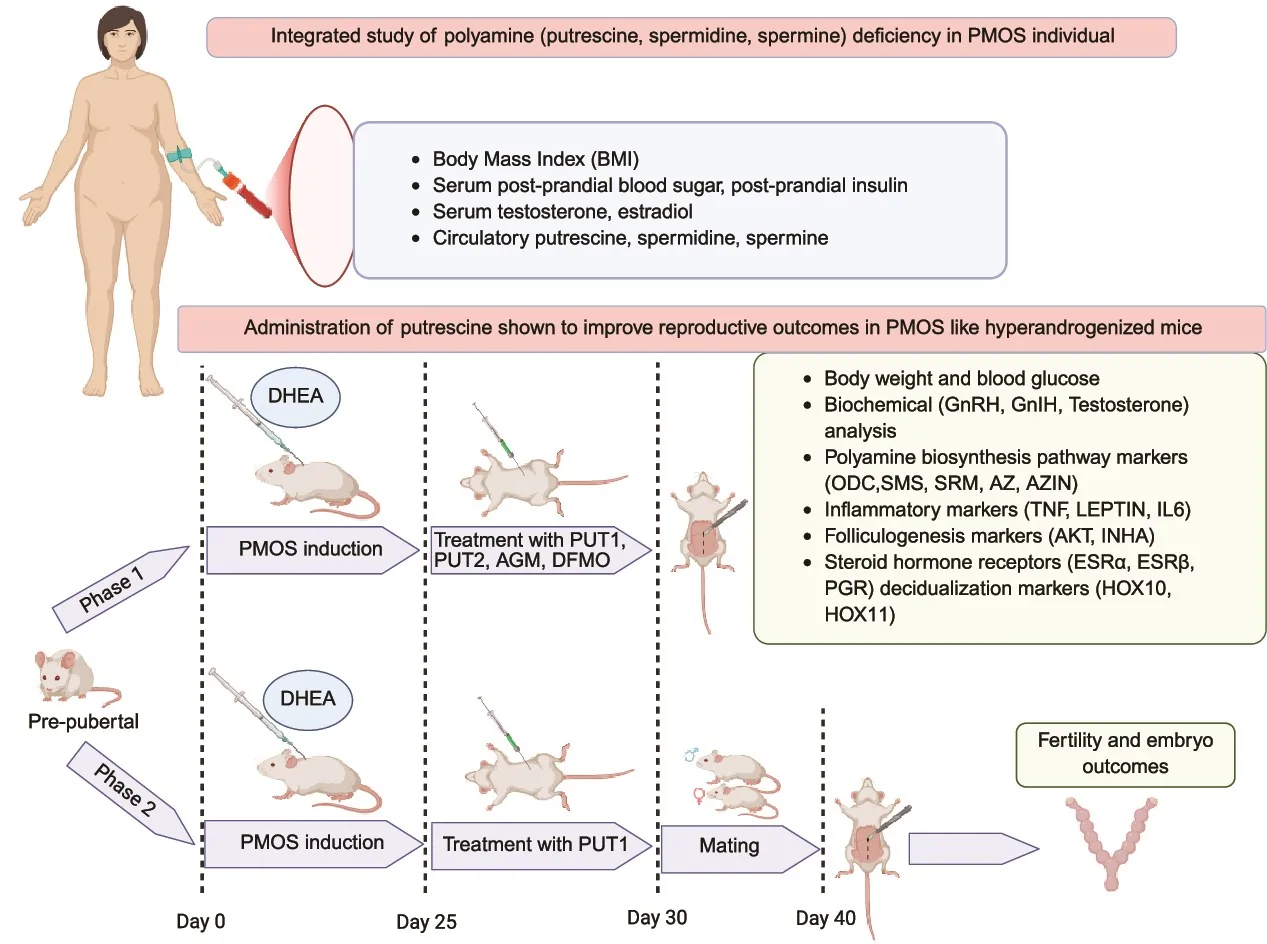

Created with BioRender.com

## Introduction

Gonadotropin-releasing hormone (GnRH) is the master regulator of mammalian reproduction by regulating the release of gonadotropins and forming an essential part of the hypothalamo-hypophyseal-gonadal (HPG) axis [1]. The reactivation of the HPG axis resulted in the onset of puberty [2]. This process is regulated by the delicate balance of the KNDy neuronal network. This network initiates pulsatile GnRH release, which is vital for neuroendocrine maturation and the development of reproductive capacity [3]. GnRH synthesis and release must be controlled to maintain an individual’s normal reproductive physiology.

Whenever an uncontrolled GnRH secretion occurs, several endocrinopathies have been observed, such as in polyendocrine metabolic ovarian syndrome (PMOS) [4]. In PMOS, pulsatile GnRH secretion gets interrupted, leading to increased GnRH secretion and an increased LH/FSH ratio from the pituitary, altering the overall HPG axis [1].

PMOS is a common metabolic syndrome affecting women of reproductive age. Its exact cause is unknown, but several factors, such as hyperandrogenism, insulin resistance, ovulatory dysfunction, and lifestyle, are associated with its development [5, 6]. The prevalence of this disorder is ~6–20% of women worldwide [7]. In the southern part of India, ~6.8% of women are diagnosed with PMOS [8]. Based on Rotterdam 2003 diagnostic criteria, a patient to be diagnosed with PMOS must have any two of these: polycyst in the ovary, hyperandrogenism, and anovulation [9].

Management of PMOS has not been well defined till this day. Metformin is currently the leading choice to manage infertility in PMOS [10]. However, its usage has some severe consequences, such as nausea, increased flatulence, gastrointestinal irritation, diarrhoea, and cramps, and one of the most potent effects is lactic acidosis observed in some individuals. Also, some combined oral contraceptive (COC) pills have been used for several years to treat individuals with PMOS.

A drug reported to be used in managing hirsutism, a condition with increased androgen levels common among PMOS patients, is eflornithine, also called α-difluoromethylornithine or DFMO. This drug is an inhibitor of the polyamine pathway [11].

Polyamines are small, aliphatic, positively charged biogenic amines found in all living organisms. In mammals, polyamines are synthesized de novo from amino acids such as arginine, methionine, and proline via L-ornithine, which is released into the blood circulation after intestinal absorption. Polyamine production and uptake from the extracellular space are controlled by proteins called antizymes and antizyme inhibitors. Polyamines have been widely reported to control the reproduction process, including embryo development and testicular and ovarian functions [12, 13]. Studies have shown elevated activity of ODC1, a rate-limiting enzyme in polyamine metabolism, during the prepubertal period in mice and rabbits [14]. Multiple reports have shown the effect of polyamines on embryogenesis and implantation [15, 16]. Studies have also shown that polyamines regulate the secretion of GnRH from the hypothalamus in neonatal rats [17], as well as regulate ovarian function in female rats by modulating GnRH [18]. Recently, polyamines have been shown to regulate the HPG axis in adult female rats, mainly by modulating the hypothalamic GnRH and the gonadotropins [19]. Recent evidence indicates that young, immature mice treated with polyamines exhibit premature folliculogenesis and an early onset of puberty, suggesting that polyamine supplementation may have therapeutic potential to improve fertility and restore reproductive function in individuals facing reproductive challenges [20]. Polyamines have also been shown to be linked with several metabolic disorders, such as childhood obesity [21] and type II diabetes [22]. Further, another study revealed that the polyamine biosynthesis pathway is altered in metabolic dysfunction-associated steatohepatitis, which leads to the expression of metabolic dysfunction-associated steatotic liver disease markers in hepatic tissue [23]. Studies have found that daily administration of high doses of polyamines can reduce body weight and adiposity in diet-induced obesity models. Additionally, this approach positively affects glucose homeostasis, indicating that exogenous polyamines may significantly impact lipid and glucose-metabolizing organs, such as the liver and fatty tissue [24, 25].

Given the established link between polyamines and metabolic disorders, their role in regulating the HPG axis, and the therapeutic use of an anti-polyamine drug (DFMO) for a PMOS-associated condition, a potential correlation between PMOS pathophysiology and polyamine metabolism warrants investigation.

Crucially, the scenario of natural polyamines in PMOS patients is not yet known. Therefore, to comprehensively understand the intricate relationship between PMOS and polyamine metabolism, it is essential to first determine the status of these polyamines in patients with PMOS.

Considering these insights, the present study hypothesizes that there may be an unexplored correlation between PMOS and polyamines. The present study aims to elucidate the effect of polyamines and their inhibitor (DFMO) in a PMOS-induced hyperandrogenized mouse model.

## Materials and methods

### Patient recruitment, sample collection, and clinical analysis

The research involved a group of 60 female participants, comprising 30 with PMOS and 30 control subjects. These individuals were recruited from the Saraswathi Speciality Clinic located in Bangalore, India. All research protocols received approval from the Institutional Ethics Committee of Padmashree Group of Institutions, Padmashree Campus, Bangalore, India, under the approval number PIEC/MLT/01/2022/AMD/1. The research procedures were carried out in strict compliance with applicable guidelines and regulations. The inclusion criteria for the study required participants to be female, aged between 18 and 40 years, diagnosed with PMOS, and to have signed informed consent forms. The exclusion criteria included (1) not meeting the above inclusion criteria; (2) having used hormonal medications (such as contraceptives, ovulation stimulators, and glucocorticoid drugs) within the preceding month; (3) being unable to fully comply with the research plan due to pregnancy, a history of infectious diseases, mental health conditions, or other issues. The diagnosis of PMOS was based on the Rotterdam criteria set by the European Society of Human Reproduction and Embryology (ESHRE) (revised 2003) [9], which necessitates the fulfilment of at least two of the following three criteria: (1) Oligo- and/or anovulation; (2) Clinical and/or biochemical evidence of hyperandrogenism; (3) Ovarian polycystic changes, as determined by ultrasound showing ≥12 follicles measuring 2–9 mm in diameter in one ovary, and/or an ovarian volume of ≥10 mL.

BMI was measured and a whole blood sample (5 mL) was taken between 08:00 and 10:00 a.m. after an overnight fast of 8–10 h and 2 mL were preserved in an EDTA tube in −80°C freezer for the assessment of polyamine levels, and the remaining were used to measure total testosterone (Roche, Cat No. 07027915190), post-prandial insulin (Roche, Cat No. 07027559190), estradiol (Roche, Cat No. 06656021190), and post-prandial blood sugar (Roche, Cat No. 04404483190) as per manufacture’s protocol. For postprandial biochemical assessments, blood samples were obtained within a standardized timeframe of 1 h 30 min to 2 h following meal intake across all study groups.

The sample size was determined by doing power analysis. To obtain the statistical power of the study, a post hoc power analysis was conducted using G^*^Power for several variables, including BMI, PP-insulin, PPBS, testosterone, putrescine, spermidine, and spermine, with a sample size of n = 30 per group. The observed effect sizes were as follows: Cohen’s d = 4.76 for BMI, Cohen’s d = 3.03 for PP-insulin, Cohen’s d = 3.45 for PPBS, Cohen’s d = 3.39 for testosterone, Cohen’s d = 14.65 for putrescine, Cohen’s d = 4.01 for spermidine, and Cohen’s d = 6.81 for spermine. The power analysis confirms that, with a sample size of n = 30 per group and an alpha level of 0.05, the study achieved a statistical power of greater than 0.99 to detect differences for all the variables.

### Analysis of free polyamines in whole blood

The total free polyamine (putrescine, spermidine, and spermine) content in whole blood was determined by HPLC using a slightly modified protocol mentioned [26]. In brief, 500 μL of whole blood was mixed with 40 μL of 70% perchloric acid and incubated at 4°C for 30 min. The mixture was then centrifuged at 15 000 × g for 10 min to isolate the supernatant. A volume of 100 μL was taken from this supernatant and combined with heptane diamine as an internal standard, followed by the addition of saturated sodium carbonate to adjust the pH to ~10. To initiate dansylation, 100 μL of freshly prepared dansyl chloride (20 mg/mL in acetone) was added to the solution. This mixture was incubated at 60°C in the dark for 1 h. The reaction was halted by introducing L-asparagine (20 mg/mL) and incubating at 60°C for an additional 30 min. The next step was to remove the acetone solvent by vacuum evaporation. Dansylated polyamines (PAs) were extracted by adding 400 μL of toluene, and the organic phase containing the dansyl-PAs was transferred to a new tube. This was followed by concentration using a vacuum concentrator, after which the samples were re-dissolved in 1 mL of methanol. For HPLC analysis, 500 μL of the prepared sample was taken into a Schimadzu system, employing a gradient elution from 40% to 100% acetonitrile in a 10 mM heptane sulfonic acid buffer (pH 3.4) using a reverse-phase C18 column. The injection volume was fixed at 10 μL, with a flow rate kept at 1 mL/min. A standard curve was created to quantify the concentrations of individual PAs found in the samples. The Shimadzu RF-20A fluorescent detector (lower detection limit in fg amounts) was used to detect the dansyl-PAs. The excitation and emission wavelengths used were 340 and 515 nm, respectively. A standard curve was generated to quantify the concentrations of individual PAs found in the samples.

### Animals and experimental design

For this study, 22-day-old pre-pubertal female Balb/c mice were used. Mice were housed for one week prior to the start of the experiment to acclimate them to the animal house environment. The animal experimentation work was planned and executed twice, once in NIRRCH (phase I) and the second in BITS Pilani K K Birla Goa Campus (phase II), as shown in the graphical abstract (Created in BioRender. Das, S. (2026) https://BioRender.com/yklagfk). Both studies were approved by the institution’s animal care and ethical committee (Birla Institute of Technology and Sciences Pilani, K. K. Birla Goa Campus; and ICMR-NIRRCH, Mumbai). Mice were housed in groups of three to four per cage under standard laboratory conditions (12 h of daylight/12 h of darkness, 25°C, and free access to rodent feed and water).

In Phase I, mice were randomly assigned to six groups (n = 4; body weight, 8–13 g). Group 1 (control), group 2 (PMOS), group 3 (PMOS+PUT1), group 4 (PMOS+PUT2), group 5 (PMOS+AGM), and group 6 (PMOS+DFMO). All the mice except group 1 were subcutaneously injected with DHEA (6 mg/100 g body weight/ day, dissolved in 0.01 ml of 95% ethanol and mixed with 0.09 ml sesame oil; Tokyo Chemical Industry Co., Ltd., Cat No. D0044) for the next 25 days, as mentioned earlier [27]. For thirty days, Group 1 was subcutaneously injected with a vehicle (0.01 mL of 95% ethanol mixed with 0.09 mL sesame oil). After completion of DHEA treatment, group 2 animals were kept alive till day 30, group 3 was injected with putrescine dose1 (PUT1) (150 ug/250 g body weight/day; Sigma-Aldrich. Ct No. P5780-5G), group 4 with putrescine dose-2 (PUT2) (250 ug/250 g body weight/day), group 5 with agmatine (AGM) (250 ug/250 g body weight/ day; Sigma-Aldrich. Cat No. A7127-1G), and group 6 with DFMO (100 mg/kg body weight/ day; Tokyo Chemical Industry Co. LTD. Cat No. D0044) for the next 5 days. All the polyamines and DFMO doses were selected based on a previous study [28–30]. After completion of the treatment, all the mice were sacrificed, and blood, hypothalamus, ovary, and uterus were collected for analysis of hormonal parameters, gene and protein expression, and morphological studies.

In Phase II, based on Phase I data, three experimental groups were established to investigate the efficacy of a selected dose of putrescine on the prevalence of successful pregnancy in PMOS-like hyperandrogenized mice (n = 4, body weight 8–13 g). Group 1 (control), group 2 (PMOS), and group 3 (PMOS+PUT1). PMOS induction and putrescine treatment were performed in the same manner as phase I. After completion of the PUT1 dose, mating was set up and confirmed by observing vaginal plug formation, which is considered day 1 of pregnancy. After 10 days of pregnancy, mice were sacrificed, and uterine images were taken.

### Body weight

Body weight was measured every five days from the start of DHEA treatment (Day 1) to the end of polyamine and DFMO treatment (Day 30) for all groups.

### Blood glucose level measurement

After completing the scheduled treatment, the mice were fasted for 8 h before obtaining blood from the tail vein to measure blood glucose using a glucometer (Gluco one, Code: A11).

### Biochemical analysis

Before sacrifice, blood was collected via the retro-orbital method. Serum was then obtained and used for ELISA. The levels of total GnRH were measured using a Mouse GnRH ELISA Kit (CUSABIO, Cat No. CSB-E08152m). The detection range is 7.8–500 pg/mL, with a sensitivity of 1.95 pg/mL. The levels of total GnIH were measured using a Mouse Gonadotropin Inhibitory Hormone (GnIH) ELISA Kit from MyBioSource (Cat No. MBS9368212). The detection range is 31.2–1000 pg/mL, and the sensitivity is 5.0 pg/mL. Finally, total testosterone levels were measured using a Mouse Testosterone ELISA kit (Cat. No. E0260Mo) by Bioassay Technology Laboratory. The detection range is 0.5 nmol/L to 100 nmol/L, with a sensitivity of 0.25 nmol/L. All biochemical analyses were performed according to the manufacturing protocol.

### Histological analysis

After dissection, the left ovary and uterus were removed from each mouse and immediately fixed in 4% paraformaldehyde overnight in 0.01 M sodium phosphate buffer. Fixed tissues were dehydrated in 70% alcohol, followed by xylene to ensure proper dehydration, and then embedded in paraffin wax. Embedded tissues were sectioned at 5 μm using a Microtome (Leica, RM2125 RTS). Sections were placed on slides and allowed to dry. According to the manufacturing protocol, Haematoxylin (Himedia, Cat. No. SO14-125ML) and eosin (Himedia, Cat. No. SO07-125ML) staining were performed. Images were taken with a compound light microscope (Leica, DM2000LED).

### Morphometric analysis of the ovary and uterus

Sections of the left ovary were taken from the entire organ semi-serially. Four equally spaced sections, stained with H&E, were randomly selected in each ovary from each animal (four mice/group), resulting in a total of 16 replicates in the experimental group for each parameter assessed. The count of healthy/atretic follicles was conducted. To identify and classify healthy follicles, the criteria outlined by Hirshfield et al [31] were employed: Class I (small preantral follicles with a diameter <90 μm); Class II (large preantral follicles with a diameter between 91 and 260 μm); Class III (small antral follicles with a diameter between 261 and 350 μm); Class IV (medium-sized antral follicles, 351–430 μm); Class V (large antral follicles with a diameter between 31 and 490 μm); Class VI (mature or Graafian follicles with a diameter >491 μm). Simultaneously, the number of visible cysts in the ovaries of mice was also counted to determine the status of the PMOS condition.

Morphometric evaluation of the uterus was conducted utilizing ImageJ software. The thicknesses of the myometrium, endometrium, and the height of the luminal epithelium were assessed in transverse sections of the uterus. For every animal (four mice per group), four measurements were taken at random locations across at least four non-consecutive sections to account for variability within the organ. These measurements were then averaged to produce a single mean value for each animal, which was used for subsequent statistical comparisons between groups.

### Toxicity assay

Collected serum was used to measure the toxicological effect of PUT1 (150 μg/250 g body weight/day) compared to the control in mice. To do so, the present study measured the concentration of alkaline phosphatase (ALP) (Coral Clinical Systems, Cat. No.: 1102020103), sensitivity 7 U/L, detection range 7–700 U/L, coefficient of variation 1.39%; SGOT (Coral Clinical Systems, Cat. No.: 1102200075), sensitivity 8.6 U/L, detection range 8.6–500 U/L, coefficient of variation 4.76%; and SGPT (Coral Clinical Systems, Cat. No.: 1102210075), sensitivity 3.8 U/L, detection range 3.8–500 U/L, coefficient of variation 5.22% followed by the kit method.

### RNA extraction and quantitative real-time PCR

Immediately following surgical excision, tissue samples were flash-frozen in liquid nitrogen and subsequently stored at −80°C to maintain RNA stability and prevent degradation. Each mouse’s right ovary, uterus, and hypothalamus were used for RNA extraction. RNA extraction and quantitative real-time PCR (qRT-PCR) assays were performed according to the kit manuals, respectively. RNA extraction was done using TRIzol reagent (Ambion by Life Technologies, USA, Cat. No. 15596026), and RNA was quantified using a NanoDrop spectrophotometer (Thermo Scientific). Genomic DNA was removed from the samples by treating them with DNase I (Thermo Scientific, Cat. No. EN0521) according to the manufacturer’s protocol. Using the First Strand cDNA Synthesis Kit (Thermo Scientific, Cat. No. K1622), 1 μg of RNA was used for cDNA synthesis. qPCR analysis was performed using Sybr-green 2X PCR master mix (Bio-Rad, Cat. 1725275). The expression level was analysed using the Agilent Technologies AriaMx Real-time PCR system. All quantitative polymerase chain reactions (qPCRs) were performed according to the manufacturer’s guidelines in a final volume of 20 μL. Each sample’s reaction was performed in triplicate, and the average value was normalized to the β-actin housekeeping gene, which showed stable expression. Earlier, β-actin had been reported to be used as a housekeeping gene in the ovary and uterus in a PMOS mouse model study [32, 33]. The expression level variations were measured using the 2^-ΔΔCT^ method. Details of primers are listed in (Supplementary Table S1). A single amplicon product and a single melt curve peak were observed for all primer pairs.

### Immunohistochemistry

The standard IHC protocol was followed to localize the proteins on tissue sections [34]. Sections were deparaffinized; endogenous peroxidase was blocked with 0.3% H_2_O_2_ in methanol for 30 min. Subsequently, the slides were washed in 0.01 M PBS and further blocked by 5% bovine serum albumin to reduce non-specific staining, followed by overnight incubation at 4°C with rabbit polyclonal antibody to aromatase (CYP19A1) (dilution 1:200; Invitrogen, Cat. No. PA1-16532), a monoclonal antibody to adiponectin (ADIPOQ) (dilution 1:20; Invitrogen, Cat. No. 701148), and a monoclonal antibody to follicular stimulating hormone receptor (FSHR) (dilution 1:200, Invitrogen, Cat. No. PA5–50963). A 1× TBST wash was performed three times for 5 min each to remove unbound antibodies. Sections were then incubated at RT for 60 mins with a biotinylated mouse anti-rabbit IgG (diluted 1:300) streptavidin-biotin horseradish peroxidase tagged complex. The unbound excess antibodies were washed three times for 5 min with 1× TBST wash. The bound complex was made visible by reaction with 3,3′-diaminobenzidine tetrahydrochloride (Roche, Cat. No. 11718096001) in 0.05 M Tris pH 7.6 and 0.1% H_2_O_2_ for 7–8 min at room temperature. A minimum of five sections was examined for each sample. Representative tissue sections were viewed and photographed under a light microscope with the nucleus counter-stained by haematoxylin (Himedia, Cat. No. SO14-125ML). Quantification of the expression of the mentioned proteins was performed using ImageJ. There was no such non-specific expression observed in the NTC section.

### Immunofluorescence

Fixed ovarian and uterine tissues were processed for an immunofluorescence assay using a standard protocol. Initially, tissues were deparaffinized with xylene and then rehydrated through a series of ethanol dilutions. Following a 1-h blocking step at room temperature, the tissues were incubated overnight at 4°C with primary antibodies: ODC1 (1:200, Invitrogen PA5-21362), SMS (1:100, Invitrogen MA5-44855), SRM (1:100, Invitrogen PA5-102895), ESRα (1:200, Invitrogen MA1-80216), ESRβ (1:200, Invitrogen PA1-311), and PGR (1:200, Invitrogen MA1-411). Unbound antibodies were removed by washing with 1× TBST buffer. The tissues were then exposed to secondary antibodies for 1 h at room temperature: Goat anti-Rabbit IgG Cy3 (1:1000, Abcam ab6939) and Goat anti-mouse IgG FITC (1:1000, Sigma-Aldrich F0257). The tissues were counterstained with DAPI for 10 min at room temperature and mounted using an appropriate medium, with cover slips sealed securely. To verify antibody specificity, negative controls lacking primary antibodies were included (data not shown). The slides were examined using a confocal microscope (Olympus Corporation FV3000), with images captured at both 10× and 40× magnification for detailed analysis. Quantification of the expression of the mentioned proteins was performed using ImageJ. There was no such non-specific expression observed in the NTC section.

### Statistical analysis

Statistical analysis was performed using GraphPad Prism. Data are presented as mean ± SEM. Before proceeding with parametric analysis, the normality of the data distribution was confirmed using the Shapiro–Wilk test (*P* > 0.05), and the equality of variances was verified using the Brown-Forsythe test. Since the data met the assumptions for parametric testing, comparisons between two groups were performed using Independent-Samples t-tests, and multiple-group comparisons were conducted using one-way ANOVA followed by Tukey’s post hoc test. Two-way ANOVA with Tukey’s post hoc test was used to analyse body weight measurement data. A *P* < 0.05 was considered statistically significant.

## Results

### Biochemical and hormonal dynamics in patients with PMOS

**Figure 1:**
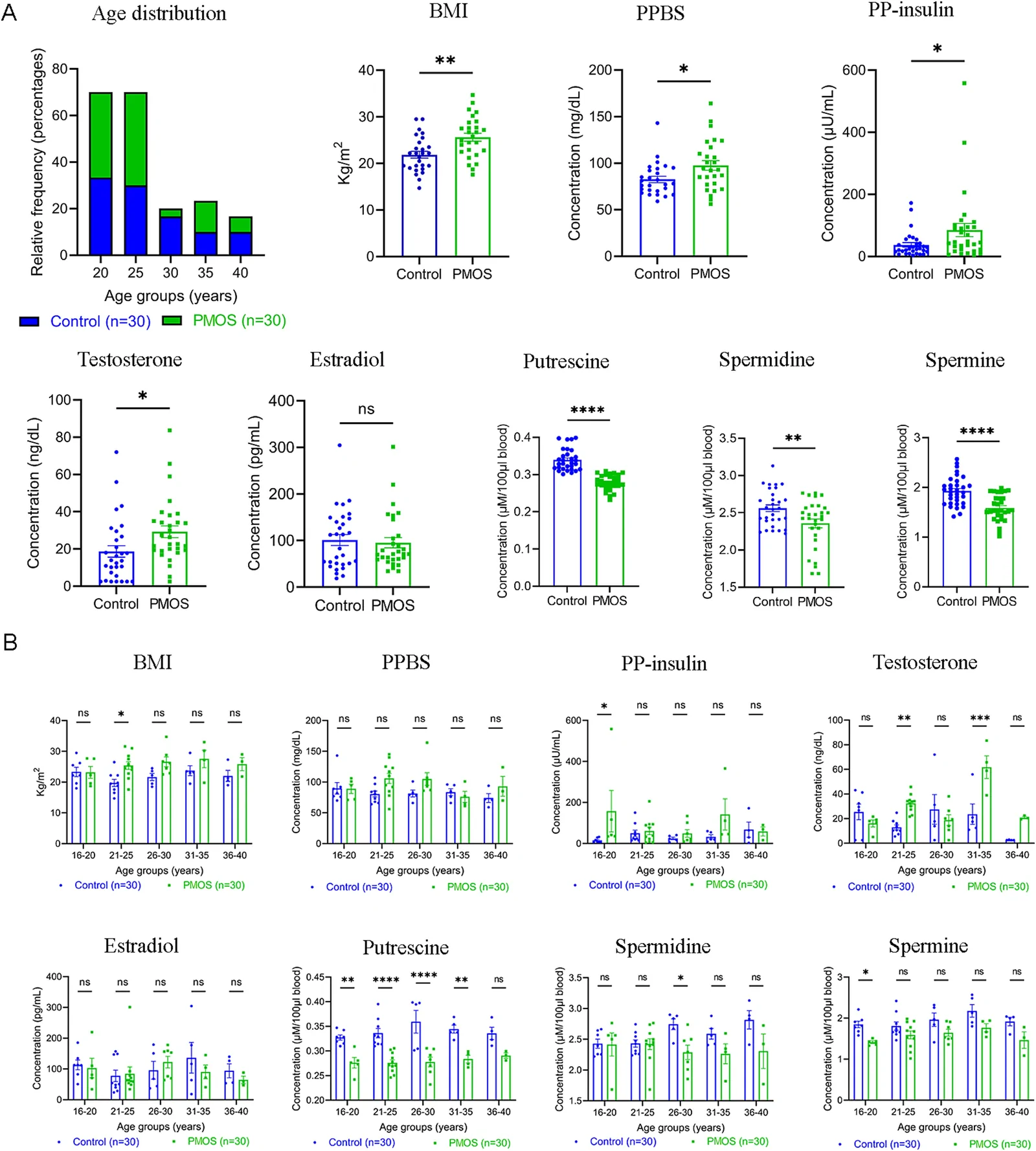
(A) The bar diagrammatic representations showing the age distribution of the participating population and modulation in biochemical parameters such as body mass index (BMI), post-prandial sugar (PPBS), post-prandial insulin (PP-insulin), Testosterone, and Estradiol in control vs PMOS individuals. The bar plots also present the different circulatory polyamines such as putrescine, spermidine, and spermine in control (n = 30) vs PMOS (n = 30). All values are represented as mean ± S.E.M; n = 30 per group; ns = *P* > 0.05, ^*^P < 0.05, ^**^P < 0.01, ^****^P < 0.0001. (B) Bar plots represent the modulation of biochemical parameters (BMI, PPBS, PP-insulin, testosterone, Estradiol), and polyamines (putrescine, spermidine, spermine) in Control (n = 30) and PMOS (n = 30) among the following age groups: 16–20 years, 21–25 years, 26–30 years, 31–35 years, and 36–40 years. All values are represented as mean ± S.E.M. ns = *P* > 0.05, ^*^P < 0.05, ^**^P < 0.01, ^***^P < 0.001, ^****^P < 0.0001.

The age distribution (Figure 1A) indicated that the 16–20, 21–25, and 36–40 age groups had nearly equal numbers of participants. Whereas, the 26–30 age group had more participants in the control group than those with PMOS, and the 31–35 age group had fewer control participants than those with PMOS. Overall, the average age of participants did not differ significantly between the control (26.47 ± 6.5966 years) and PMOS (25.67 ± 6.0018 years) groups (Supplementary Table S2). Through assessment of biochemical and hormonal parameters, it was established that patients with PMOS exhibited significantly elevated body mass index (BMI) and increased blood pp-insulin and pp-glucose levels (Figure 1A). Furthermore, it was noted that free testosterone concentrations were markedly higher in PMOS patients in comparison to healthy female controls, but there was no significant difference in estradiol levels (Figure 1A). In the 21–25 age group, individuals with PMOS have a significantly higher BMI compared to the control group. In other age groups, there is a noticeable upward trend in BMI among those with PMOS (Figure 1B). Additionally, the PPBS levels in individuals with PMOS also show an increasing trend across all age groups (Figure 1B). Notably, PP-insulin levels in the 16–20 age group of PMOS patients are significantly higher than those in the control group (Figure 1B). Furthermore, testosterone levels in the 21–25 and 31–35 age groups of individuals with PMOS are significantly elevated compared to controls (Figure 1B). However, no significant differences in estradiol levels are observed across age groups when comparing individuals with PMOS to the control group (Figure 1B).

### Polyamine dynamics in patients with PMOS

An HPLC analysis of blood samples from patients with PMOS demonstrated significantly reduced levels of circulating polyamines—specifically putrescine, spermidine, and spermine—compared to healthy controls (Figure 1A; Supplementary Table S2).

In the 16–20, 21–25, 26–30, and 31–35 years age groups, the circulatory putrescine level is significantly lower in PMOS than in controls (Figure 1B). Spermidine level is significantly lower in the 26–30 years age group in PMOS compared to controls; in the 31–35 and 36–40 years age groups, levels are also noticeably lower in PMOS (Figure 1B). Spermine levels are significantly lower in the 16–20-year age group in PMOS compared to controls; in all other age groups, levels are also noticeably lower in PMOS (Figure 1B).

### Polyamines regulate body weight in PMOS mice

**Figure 2:**
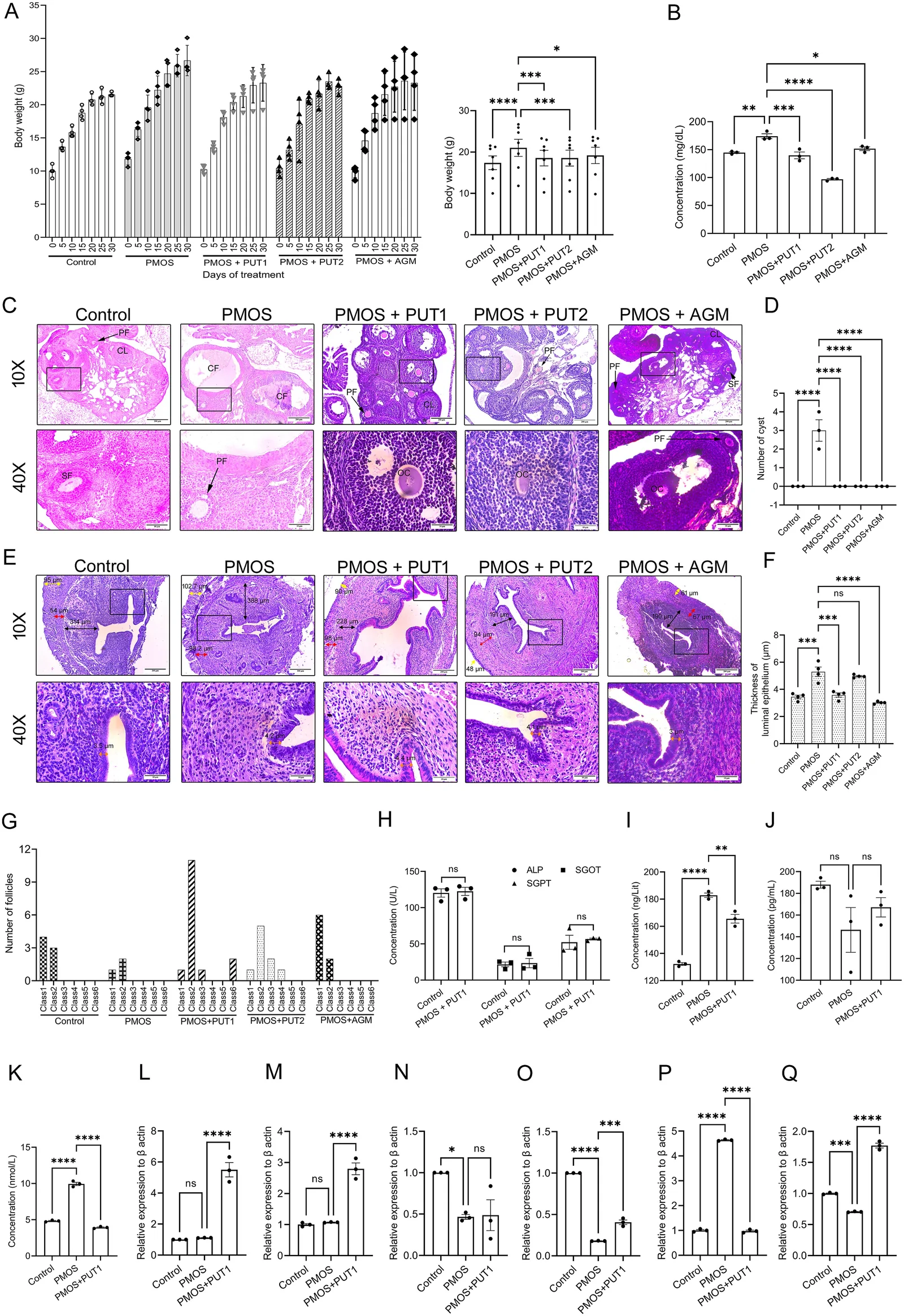
(A) Graphical representation of the measurements of body weights of mice groups consisting of control mice, DHEA-treated PMOS-like hyperandrogenized mice, and PMOS-like hyperandrogenized mice treated with PUT1, PUT2, and AGM, respectively, at an interval of 5 days. The left image shows how the individual body weights of every mouse in each group (n = 4) changed at every 5-day interval. The right image represents the comparison between the mean body weight of all mice across all groups (n = 4). The groups PMOS+PUT1, PMOS+PUT2, and PMOS+AGM were treated with their respective polyamines for 5 days after 25 days of DHEA treatment. The data obtained from days 1 to 30, in 5-day intervals for all mice (n = 4) from each group, were utilized to determine statistical significance. Results were expressed as mean ± S.E.M. Two-way ANOVA with Tukey’s post hoc test was used to analyse body weight measurement data. ^*^P < 0.05, ^***^P < 0.001, ^****^P < 0.0001, ns *P* > 0.05. (B) The bar diagrammatic representation at the panel shows the blood glucose levels of the different groups of mice at day 30. All value represented as mean ± S.E.M; n = 3; ^****^P < 0.0001, ^***^P < 0.001, ^**^P < 0.01, ^*^P < 0.05. (C) The H&E-stained histological sections of representative ovaries from each group of mice. The upper panel consists of microscopic images at 10× magnification, and the lower panel represents the 40× magnification of the portion marked with a black rectangle of the respective images at the upper panel. Here, PF means primary follicle, SF means secondary follicle, CL means corpus luteum, CF means cystic follicle, and OC means oocyte. (D) Graphical representation showing the number of cysts present in the ovaries of different groups of mice at day 30. (E) The H&E-stained histological sections of the representative uterus from each group of mice show changes in perimetrium (yellow arrow), myometrium (red arrow), and endometrium (black arrow). The upper panel represents microscopic images at 10× magnification, and the lower panel represents 40× magnification of the portions marked with black rectangles at the respective upper panel images. The scale bar in the 10× magnification images represents 200 μm, and the scale bar in the 40× magnification images represents 10 μm. (F) Graphical representation showing the changes in the thickness of the luminal epithelium of uteri from different groups of mice. All values are represented as mean ± S.E.M; n = 4; ^****^P < 0.0001, ^***^P < 0.001, ns = *P* > 0.05. (G) Graphical representation showing different classes of follicles in the ovaries of control mice, untreated PMOS-like mice, and PMOS-like mice treated with PUT1, PUT2, and AGM. All values are represented as mean ± S.E.M; n = 4; ^****^P < 0.0001, ^***^P < 0.001. (H) The bar plot represents the changes in the levels of ALP (alkaline phosphatase), SGOT (serum glutamic oxaloacetic transaminase), and SGPT (serum glutamic pyruvate transaminase) in the PMOS mice group, followed by treatment with a selected dose of putrescine (PUT1). All values are represented as mean ± S.E.M; n = 3; ns = *P* > 0.05. (I, J, K) The bar plots represent the changes in serum levels of GnRH, GnIH, and testosterone due to the induction of PMOS in mice and the treatment of the PMOS-induced mice group with PUT1. All values are represented as mean ± S.E.M; n = 3; ns = *P* > 0.05, ^**^P < 0.01, ^***^P < 0.001, ^****^P < 0.0001. (L, M, N, O, P, Q) The bar diagrams present the alterations in the expressions of hypothalamic genes like ODC1, SMS, Az, Azin, GnRH, and GnIH in the PMOS-induced mice group and the PMOS mice treated with PUT1. All values are represented as mean ± S.E.M; n = 3; ns = *P* > 0.05, ^*^P < 0.05, ^***^P < 0.001, ^****^P < 0.0001.

Measuring the body weight of mice after every 5-day interval (Figure 2A, left panel) revealed that the body weight of DHEA-treated (PMOS group) mice was significantly higher compared to the control group (Group: control = 17.4 g, PMOS = 21.0286 g, ^****^P < 0.0001) (Figure 2A, right panel). After treatment with polyamines and DFMO, the body weight of the PUT1 and PUT2-treated PMOS-induced mice was observed to be significantly low compared to the PMOS group (Group: PMOS = 21.0286 g, PMOS+PUT1 = 18.5429 g, and PMOS+PUT2 = 18.5335 g, ^***^P < 0.001); moreover, AGM treated mice also exhibited significantly reduced body weight compared to PMOS (Group: PMOS = 21.0286 g, PMOS+AGM = 19.1929 g, ^*^P < 0.05) (Figure 2A, right panel). No significant effect was observed in the body weight of the DFMO-treated group of mice when compared with that of the PMOS group (Supplementary Figure S1).

### Polyamines maintain blood glucose homeostasis in PMOS mice

The blood glucose level was also observed to be significantly higher in PMOS mice compared to control mice (Group: Control = 144.67 mg/dL, PMOS = 174.00 mg/dL, ^**^P < 0.01) (Figure 2B). When treated with polyamines, blood glucose level significantly decreased compared to PMOS (Group: PMOS = 174.00 mg/dL, PMOS+PUT1 = 139.67 mg/dL, ^***^P < 0.001; PMOS+PUT2 = 96.79 mg/dL, ^****^P < 0.0001; PMOS+AGM = 151.667 mg/dL, ^*^P < 0.05; PMOS+DFMO = 73.8378 mg/dL, ^****^P < 0.0001) (Figure 2B, Supplementary Figure S1).

### Polyamines improve morphological changes in the ovaries and uterus of PMOS mice

Follicles at different stages, such as primary, secondary, mature, corpus lutetium, and oocyte, were observed in the control mice ovary, along with normal theca and granulosa cells (Figure 2C). In the PMOS ovary, different stages of the follicle are absent; instead, an abundance of fluid fills cystic follicles, which are evident. The granulosa layers were observed to be mostly abolished. A thick layer of theca cells was observed with an immature oocyte (Figure 2C). After treatment with PUT1, PUT2, and AGM, there was a visible improvement in the number of different stages of follicles with normal granulosa and theca cells layers, PUT1 treatment caused better improvement in the ovarian morphology rather than other polyamine doses (Figure 2C). The ovaries from the DFMO-treated mice, on the other hand, showed no significant improvement (Supplementary Figure S1). Analysing and classifying the healthy follicles following the criteria of Hirshfield et al. revealed that the ovaries of PMOS-like hyperandrogenized mice showed a reduced number of both Class 1 and Class 2 follicles compared to healthy control mice. PUT1 treatment significantly increased the number of Class 2 follicles, whereas PUT2, AGM, and DFMO treatment did not alter the numbers of Class 1 or Class 2 follicles in the ovaries of PMOS-like hyperandrogenized mice. On the other hand, a few Class 3 and Class 4 follicles were evident in the ovaries of PUT2- and DFMO-treated PMOS-like hyperandrogenized mice (Figure 2D, Supplementary Figure S1). Moreover, the cysts, which were evident in the ovaries of the untreated PMOS-like hyperandrogenized mice, were not present in any of the polyamine-treated mouse groups. However, the cysts were still visible in the ovaries of the PMOS-like hyperandrogenized mice after DFMO treatment (Figure 2E, Supplementary Figure S1). The H&E-stained uterine tissue from PMOS mice exhibited a thick uterine wall and a heightened luminal epithelium layer (Figure 2F) with the accumulation of endometrial glands compared to the uterus from the control group of mice (Figure 2G). Similarly, a significantly improved luminal epithelium lining with a thin uterine wall was observed in PUT1-treated mice compared to other groups of polyamines- and DFMO-treated mice (Supplementary Figure S1).

### Toxicological effect of PUT1 on mice

There were no significant changes in serum ALP, SGOT, and SGPT concentrations in the putrescine-treated group compared with the control; thus, no toxicity was observed with the final dose of putrescine used to treat the PMOS-like hyperandrogenized mice (Figure 2H).

### Polyamine regulates endocrine parameters and androgen in PMOS mice

Since the secretion of neurohormones such as GnRH and GnIH is known to be altered in PMOS, the expression of these hormones was assessed in different groups of mice. The analysis revealed that the circulatory level of GnRH was significantly high in PMOS compared to the control (Group: control = 132.25 ng/lit, PMOS = 182.85 ng/lit, ^****^P < 0.0001) (Figure 2I). After treatment with PUT1, GnRH level was found to be significantly reduced compared to PMOS (Group: PMOS = 182.85 ng/lit, PMOS+PUT1 = 165.54 ng/lit, ^**^P < 0.01) (Figure 2I). There were no significant changes observed in the level of GnIH among all the groups (Figure 2J).

The circulatory testosterone level was significantly higher in PMOS compared to control (Group: Control = 4.825 nmol/L, PMOS = 9.945 nmol/L, ^**^P < 0.01) (Figure 2K), which is also known to be one of the key responsible factors for PMOS. Following polyamine treatment, it was significantly reduced compared to PMOS (Group: PMOS = 9.945 nmol/L, PMOS+PUT1 = 3.93 nmol/L, ^***^P < 0.001) (Figure 2K).

### Hypothalamic gene expression study

#### Exogenous putrescine elevates endogenous polyamine catabolism in the PMOS mice hypothalamus

The exogenous PUT1 treatment on PMOS hyperandrogenized mice resulted in significant upregulation of ODC1 (Group: Control = 1, PMOS = 1.11346, PMOS+PUT1 = 5.50282, ^****^P < 0.0001) (Figure 2L), and SMS (Group: Control = 1, PMOS = 1.07414, PMOS+PUT1 = 2.79878, ^****^P < 0.0001) (Figure 2M) genes in the hypothalamus compared to PMOS, no significant changes were observed between the control and PMOS groups. The expression of ornithine decarboxylase antizyme 1 (Oaz1) or antizyme 1 (Az) (Group: Control = 1, PMOS = 0.46565, ^*^P < 0.05) and antizyme inhibitor 1 (Azin) (Control = 1, PMOS = 0.18028, ^****^P < 0.0001) in the hypothalamus of PMOS mice was significantly lower than in the control. Once treated with PUT1, the expression of Azin was significantly higher compared to PMOS (PMOS = 0.18028, PMOS+PUT1 = 0.40564, ^***^P < 0.001) (Figure 2N), and there were no significant changes observed in Az between the PMOS and the PUT1-treated PMOS group (Figure 2O).

#### Exogenous putrescine regulates the expression of GnRH and GnIH in the PMOS mice hypothalamus

The expression of GnRH in the PMOS mice hypothalamus was significantly higher than control (Group: Control = 1, PMOS = 4.63752, ^****^P < 0.0001). Once treated with PUT1, the expression of GnRH was significantly down-regulated compared to PMOS (Group: PMOS = 4.63752, PMOS+PUT1 = 0.972935, ^****^P < 0.0001) (Figure 2P). This study also demonstrated that the scenario of GnIH in the hypothalamus of PMOS mice was precisely opposite to GnRH. In PMOS mice, hypothalamus expression of GnIH was significantly down-regulated compared to control (Group: control = 1, PMOS = 0.70464, ^***^P < 0.001), and once treated with PUT1, it showed significant upregulation compared to PMOS (Group: PMOS = 0.70464, PMOS+PUT1 = 1.77228, ^****^P < 0.0001) (Figure 2Q).

#### Putrescine manages the expression of inflammatory and follicular markers in the PMOS mice ovary, and endogenous polyamine metabolism in the PMOS mice ovary

**Figure 3:**
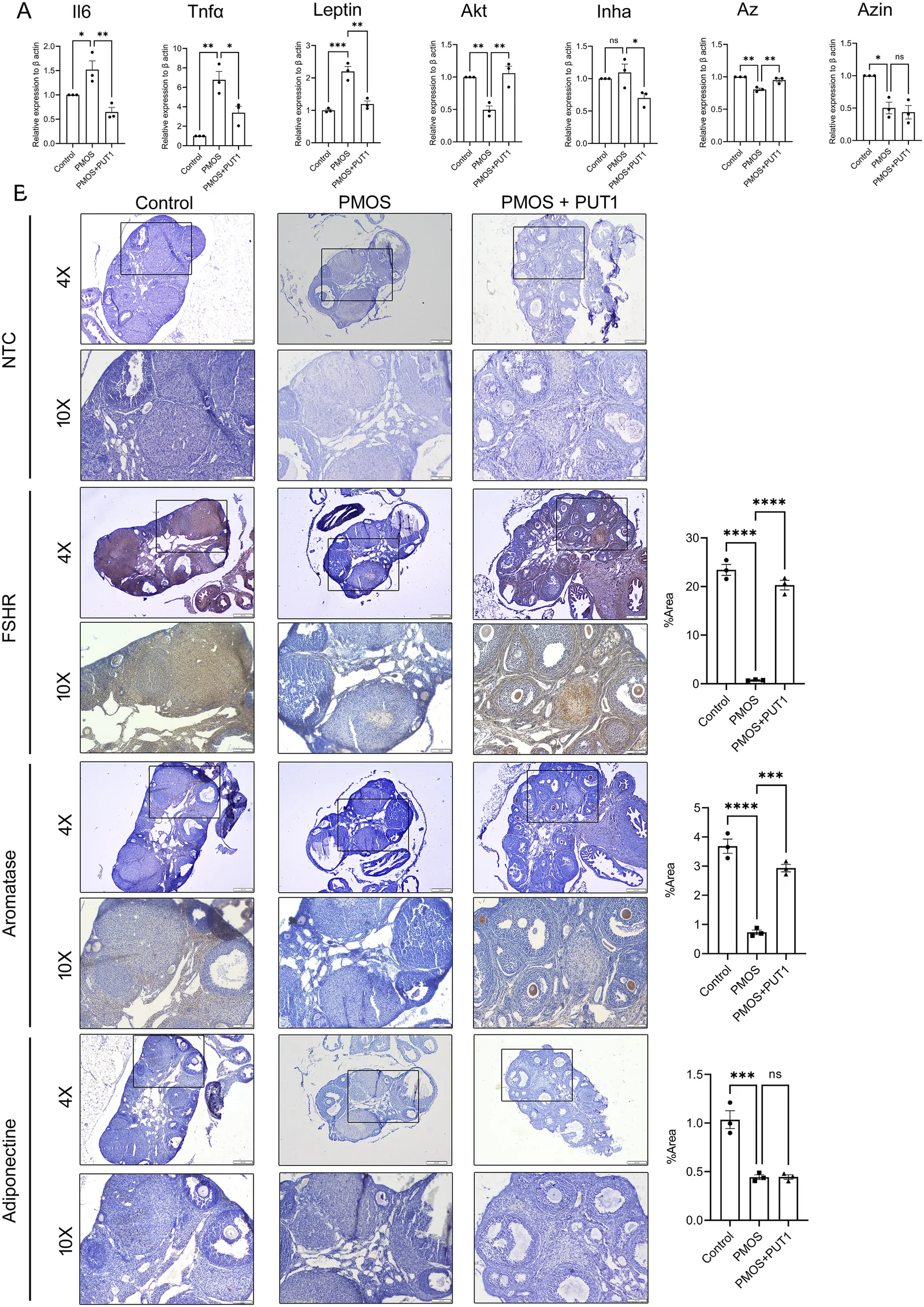
(A) The bar diagrammatic representations showing modulation in the gene level expressions of markers of inflammation, folliculogenesis, and polyamine biosynthesis pathway enzymes such as Il6, Tnfα, Leptin, Akt, Inha, Az, and Azin (from left to right) in PMOS-like hyperandrogenized mice ovary, compared with control and then the effect of PUT1 treatment on the expression level of these genes in PMOS-like hyperandrogenized mice ovary. All values are represented as mean ± S.E.M; n = 3; ^****^P < 0.0001, ^***^P < 0.001, ^**^P < 0.01, ^*^P < 0.05. (B) The microscopic images (at 4× and 10× magnifications) obtained from immunohistochemistry of histological sections of ovaries from control mice, PMOS-induced mice, and PMOS mice treated with the PUT1 groups with anti-FSHR, anti-aromatase, and anti-adiponectin antibodies. Aromatase expression throughout theca cell layer, mural granulosa cells, and corpus luteum is marked as a black arrow on a 10× row. The quantification of DAB staining intensity was performed using ImageJ software. All values are represented as mean ± S.E.M; n = 3; ^****^P < 0.0001, ^***^P < 0.001, ns = *P* > 0.05.

When the expression of the inflammatory markers were assessed, it was observed that the expressions of Tnfα (Group: Control = 1, PMOS = 6.76854 ^**^P < 0.01), Il6 (Group: Control = 1, PMOS = 1.51986 ^*^P < 0.05), Leptin (Group: Control = 1, PMOS = 2.20172 ^***^P < 0.001) were significantly up-regulated in PMOS hyperandrogenized mice compared to control (Figure 3A), once treated with PUT1, the expression of Tnfα (Group: PMOS = 6.76854, PMOS+PUT1 = 3.38418 ^*^P < 0.05), Il6 (Group: PMOS = 1.51986, PMOS+PUT1 = 0.647162 ^**^P < 0.01), and Leptin (Group: PMOS = 2.20172, PMOS+PUT1 = 1.19465 ^**^P < 0.01) shows a significant downregulation compared to PMOS. However, the follicular marker, such as Akt (Group: Control = 1, PMOS = 0.493805 ^**^P < 0.01), was also significantly down-regulated in the ovaries of PMOS hyperandrogenized mice; it was upregulated (Group: PMOS = 0.493805, PMOS+PUT1 = 1.0595 ^**^P < 0.01) once treated with PUT1. The expression of Inha (Group: Control = 1, PMOS = 1.09532, PMOS+PUT1 = 0.701717 ^*^P < 0.05) was also significantly down-regulated in PMOS hyperandrogenized mice when treated with PUT1 compared to PMOS (Figure 3A). The expression of Az (Group: Control = 1, PMOS = 0.80607, ^**^P < 0.01) and Azin (Group: Control = 1, PMOS = 0.47741, ^*^P < 0.05) was significantly lower in the PMOS mouse ovary compared to the control. Once treated with PUT1, the expression of Az (Group: PMOS = 0.80607, PMOS+PUT1 = 0.95079, ^**^P < 0.01) was significantly higher than in PMOS. There were no significant changes in Azin expression in the ovary of PUT1-treated PMOS mice compared to PMOS (Figure 3A).

#### Effect of putrescine on the translational expression of FSHR, CYP19A1, and adiponectin in the ovaries of PMOS mice

The variations in the immunostaining pattern of FSHR, CYP19A1, and ADIPOQ expression were also assessed in the ovaries of control mice, mice with PMOS, and PMOS mice treated with putrescine. In the control group, FSHR and aromatase were intensely expressed in the ovary’s primary, secondary, and tertiary follicles, oocytes, and corpus luteum parts (Figure 3B). In comparison, the expression of FSHR and aromatase was lower in the PMOS group than in the control group. However, when treated with putrescine, the immunolocalisation of FSHR and aromatase was moderate in the primary, secondary, and tertiary follicles, oocytes, and corpus luteum parts (Figure 3B). The adiponectin expression was lower in both PMOS and PUT1-treated PMOS groups compared to the control group (Figure 3B).

### Uterine gene expression study

#### Putrescine regulates the expression of steroid hormone receptors in the PMOS mice uterus

**Figure 4:**
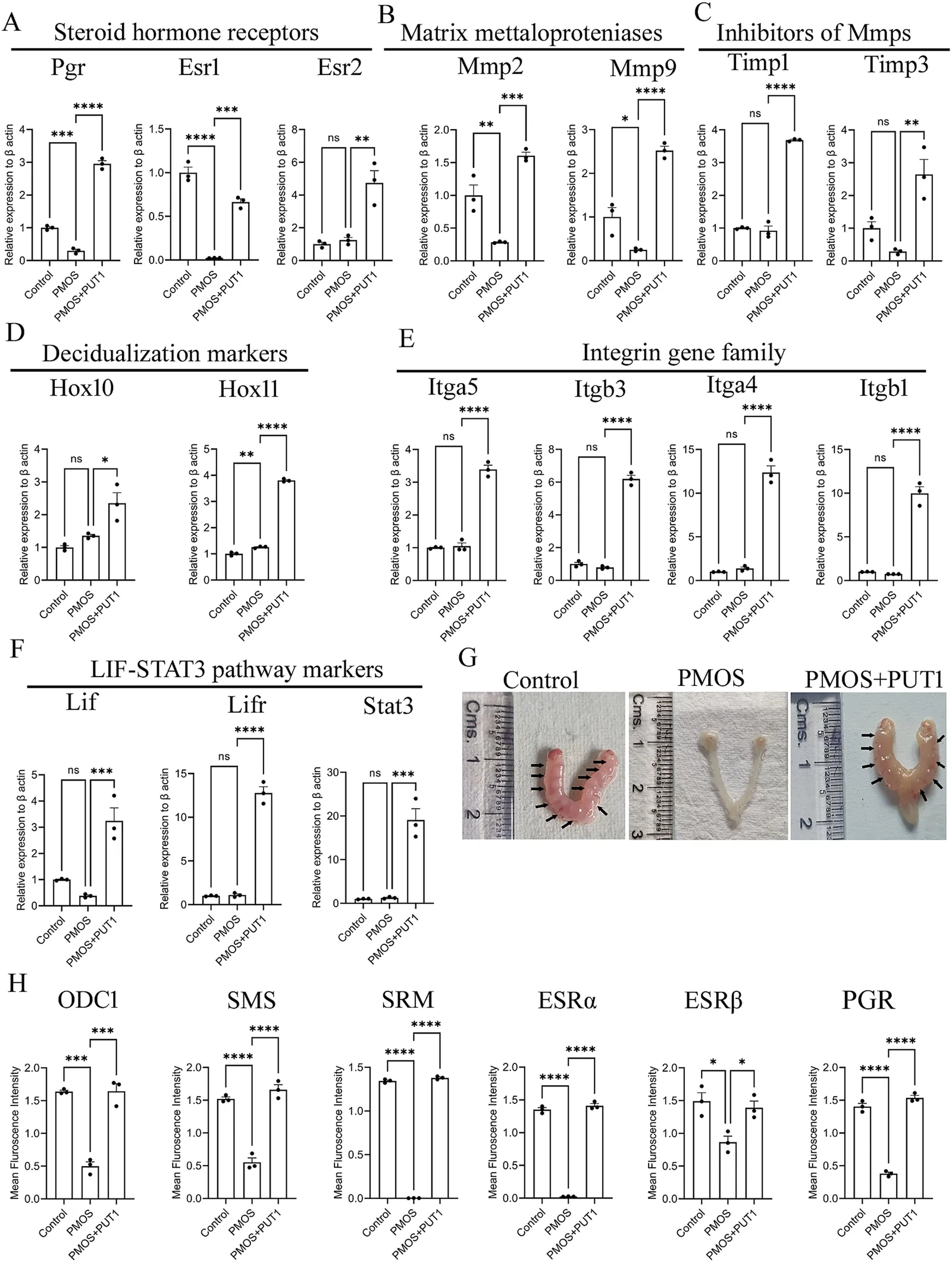
The bar diagrams representing alterations in the gene expression levels of (A) steroid hormone receptors such as PGR, ESR1, ESR2, (B) matrix metalloproteinases such as MMP2 and MMP9, (C) inhibitors of matrix metalloproteinases such as TIMP1 and TIMP3, and (D) decidualization markers HOX10 and HOX11 in the uterus of PMOS-like hyperandrogenized mice compared to control and the effect of PUT1 treatment on the expression of these genes in PMOS-like hyperandrogenized mice uterus. The effect of PUT1 on the gene expression levels of ITGA5, ITGB3, ITGA4, and ITGB1 from integrin gene family (E), as well as LIF, LIFR, and STAT3 from Lif-Stat3 pathway genes (F) on PMOS mice groups, are represented in bar diagrams. All values are represented as mean ± S.E.M; n = 3; ns = *P* > 0.05, ^****^P < 0.0001, ^***^P < 0.001, ^**^P < 0.01, ^*^P < 0.05. The photographic images in panel (G) represent the uterus of mice from control, PMOS-induced, and PUT1-treated PMOS mice groups, showing the status of implantation and pregnancy in the mouse models. The scale bars in the respective images represent measurements in centimeters. The bar diagram (H) represents the quantification of the immunofluorescence study of ODC1, SMS, SRM, ESRα, ESRβ, and PGR. All values are represented as mean ± S.E.M; n = 3; ^****^P < 0.0001, ^***^P < 0.001, ns = *P* > 0.05.

The expressions of steroid hormone receptors such as Progesterone receptor (Pgr) (Group: Control = 1, PMOS = 0.297149 ^****^P < 0.0001), Estrogen receptor1 (Esr1) (Group: Control = 1, PMOS = 0.024191^****^P < 0.0001) are significantly downregulated in PMOS compared to control, there was no significant changes were observed for Esr2 (Group: Control = 1, PMOS = 1.24765 ns *P* > 0.05), once treated with PUT1, Pgr (Group: PMOS = 0.297149, PMOS+PUT1 = 2.95411 ^****^P < 0.0001), Esr1 (Group: PMOS = 0.024191, PMOS+PUT1 = 0.66379 ^***^P < 0.001), and Esr2 (Group: PMOS = 1.24765, PMOS+PUT1 = 4.74656 ^**^P < 0.01) were significantly up-regulated in putrescine treated mice uterus compared to PMOS mice (Figure 4A).

#### Putrescine elevated the expression of matrix metalloproteinases and their inhibitor in the PMOS mice uterus

The expression of Mmp2 (Group: Control = 1, PMOS = 0.284962 ^**^P < 0.01), and Mmp9 (Group: Control = 1, PMOS = 0.249179 ^*^P < 0.05) were significantly downregulated in PMOS mice uterus compared to control (Figure 4B). The expressions of Mmp2 (Group: PMOS = 0.284962, PMOS+PUT1 = 1.60528 ^***^P < 0.001) and Mmp9 (Group: PMOS = 0.249179, PMOS+PUT1 = 2.40996 ^***^P < 0.001) were significantly up-regulated in putrescine-treated PMOS uterus compared to PMOS mice (Figure 4B). Simultaneously, the expressions of metalloproteinases (MMPs)’ inhibitors such as Timp1 (Group: PMOS = 0.923142, PMOS+PUT1 = 3.68822 ^****^P < 0.0001) and Timp3 (Group: PMOS = 0.288011, PMOS+PUT1 = 2.64541 ^**^P < 0.01) were also significantly up-regulated in putrescine-treated PMOS mice uterus, compared to PMOS mice (Figure 4C), there was no significance changes were observed between control and PMOS groups.

#### Putrescine upregulates the expression of decidualization markers in the PMOS mice uterus

The expression of decidualization markers, such as Hox11 (Group: Control = 1, PMOS = 1.2552, ^**^P < 0.01), was significantly upregulated in PMOS compared to control. However, there was no significant change in the expression of Hox10 (Group: Control = 1, PMOS = 1.35374 ns *P* > 0.05) in the PMOS uterus compared to the control (Figure 4D). Furthermore, Hox10 (Group: PMOS = 1.35374, PMOS+PUT1 = 2.35025 ^*^P < 0.05) and Hox11 (Group: PMOS = 1.2552, PMOS+PUT1 = 3.89575 ^****^P < 0.0001) were significantly up-regulated in putrescine-treated PMOS uterus compared to PMOS mice (Figure 4D).

#### Putrescine increases the expression of the integrin gene family in the PMOS mice uterus

The expressions of integrin family genes such as Itga5 (Group: Control = 1, PMOS = 1.04895, PMOS+PUT1 = 3.39097 ^****^P < 0.0001), Itgb3 (Group: Control = 1, PMOS = 0.788079, PMOS+PUT1 = 6.19489 ^****^P < 0.0001), Itga4 (Group: Control = 1, PMOS = 1.38598, PMOS+PUT1 = 12.3633 ^****^P < 0.0001), and Itgb1 (Group: Control = 1, PMOS = 0.748146, PMOS+PUT1 = 9.98639, ^****^P < 0.0001) was significantly up-regulated in putrescine-treated PMOS mice uterus compared to PMOS mice, there was no significant changes were observed in PMOS uterus compared to control (Figure 4E).

#### Putrescine elevated the expression of Lif-Stat3 signalling pathway genes in the PMOS mice uterus

It was observed that the expressions of Lif (Group: Control = 1, PMOS = 0.379207, PMOS+PUT1 = 3.24429 ^***^P < 0.001), Lifr (Group: Control = 1, PMOS = 1.10209, PMOS+PUT1 = 12.7816 ^****^P < 0.0001), and Stat3 (Group: Control = 1, PMOS = 1.26327, PMOS+PUT1 = 19.0766 ^***^P < 0.001) were significantly up-regulated in putrescine-treated PMOS mice uterus compared to PMOS mice, there was no significant change in expression was observed between control and PMOS uterus (Figure 4F).

#### Exogenous putrescine administration enhanced the endogenous polyamine catabolism in the PMOS-like hyperandrogenized mouse ovary

**Figure 5:**
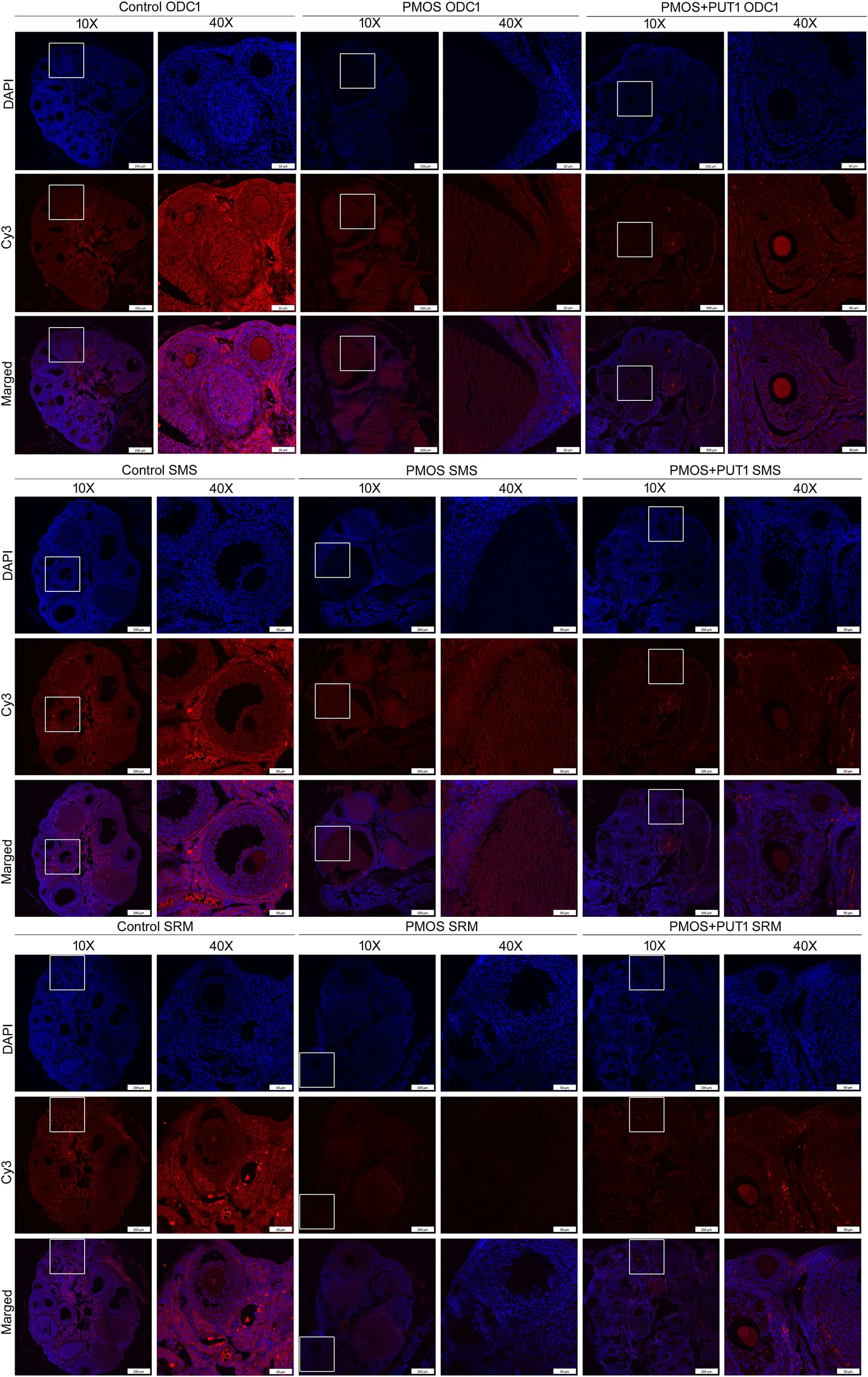
Microscopic immunofluorescence images acquired at 10× and 40× magnification illustrate histological sections of ovaries from three groups: control mice, PMOS-like hyperandrogenized mice, and PMOS-like hyperandrogenized mice treated with the PUT1. The expression of specific proteins was assessed using anti-ODC1, anti-SMS, and anti-SRM antibodies. Red fluorescence indicates the presence of the respective proteins, while the nuclear stain DAPI is represented in blue. Quantitative analysis of protein expression intensity was performed using ImageJ software.

The variation in the immunofluorescence staining patterns of ODC1, SMS, and SRM expression was measured in the Control, PMOS-like hyperandrogenized, and PUT1-treated PMOS-like hyperandrogenized groups. Notably, in the PMOS-like hyperandrogenized mice, the expression levels of ODC1, SMS, and SRM were significantly diminished in various ovarian structures, including primordial follicles, primary and secondary follicles, graafian follicles, oocytes, as well as granulosa and theca cells, when compared to the Control group (Figure 4H, Figure 5, and Supplementary Table S3). In contrast, treatment with putrescine resulted in a marked restoration of expression for these polyamine catabolism markers across multiple ovarian compartments (Figure 4H and Figure 5).

#### Exogenous putrescine treatment improves the translational expression of steroid hormone receptors (ESRα, ESRβ, and PGR) in PMOS-like hyperandrogenized mice uterus

**Figure 6:**
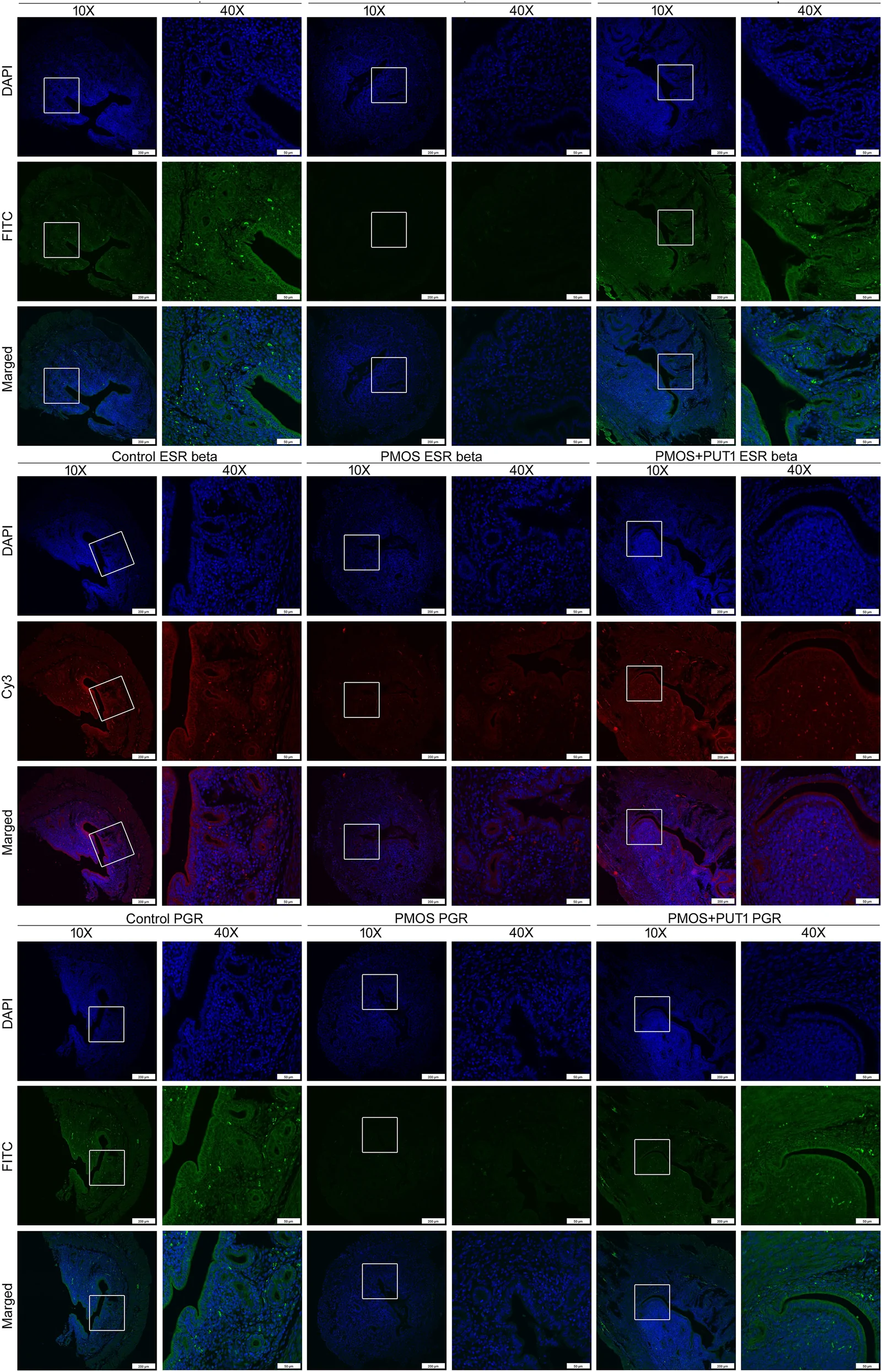
The microscopic images (at 10× and 40× magnifications) obtained from immunofluorescence of histological sections of the uterus from control mice, PMOS-like hyperandrogenized mice, and PMOS-like hyperandrogenized mice treated with the PUT1 groups with anti-ESRα, anti-ESRβ, and anti-PGR antibodies. Green fluorescence represents the expression of ESRα and PGR. Red fluorescence indicates ESRβ expression, and blue represents the nuclear stain DAPI. Quantitative analysis of protein expression intensity was performed using ImageJ software.

For successful implantation and pregnancy, the endometrium undergoes critical steroid-dependent modifications, largely regulated by the steroid hormone receptors localized in the endometrial tissue. In this study, immunofluorescence analysis demonstrated significant downregulation of ESRα, ESRβ, and PGR in the luminal and glandular epithelium, as well as in the uterine stromal compartments, in PMOS-like hyperandrogenized mice compared to the control cohort (Figure 4H, Figure 6, and Supplementary Table S4). Notably, the administration of putrescine was found to restore the expression levels of these steroid hormone receptors in the affected uterine tissues (Figure 4H, Figure 6, and Supplementary Table S4).

#### Putrescine improves the implantation scenario in the PMOS mice’s uterus

A study of implantation and zygote formation in control, PMOS, and putrescine-treated mice revealed that all control mice exhibited healthy implantation and zygote formation. In contrast, no implantation or zygote formation occurred in PMOS-like hyperandrogenized mice. Half of the PUT1-treated PMOS-like hyperandrogenized mice have shown healthy implantation in both horns, thereby achieving a 50% implantation rate in successful pregnancies. (Figure 4G).

## Discussion

### Polyamine dysregulation and the PMOS phenotype

PMOS is a chronic and incurable condition [35], has been an area of great interest to reproductive biologists for decades. Although several studies have been carried out exploring various regimens to manage and improve PMOS conditions, a reliable and unfailing treatment regimen has yet to be achieved. Therefore, this study primarily aimed to establish a therapeutic target that can be utilized to manage PMOS conditions. Moreover, polyamines, such as putrescine, spermidine, and spermine, are crucial for mammalian ovarian function. They are involved in several key ovarian processes, such as steroidogenesis, follicular development, oocyte maturation, ovarian ageing, and controlling aneuploidy in oocytes [36, 37]. Polyamine supplementation in adult female rodents has shown that it can increase hypothalamic GnRH expression and release, along with an increase in LH, FSH, and steroid hormones [19]. The positive effect of polyamines on ovarian physiology and hypothalamic GnRH release might be utilized to improve PMOS-like conditions in the ovary.

For the first time, this study has deciphered a link between polyamines and the pathophysiology of PMOS. The present work found that circulatory polyamines (putrescine, spermidine, and spermine) are significantly downregulated in PMOS patients and indicates that treating PMOS-like hyperandrogenized mice with polyamines, primarily putrescine, can improve ovarian physiology and manage PMOS-like conditions. Putrescine has been observed to ameliorate PMOS-like features in mouse models by primarily regulating hypothalamic GnRH levels and further enhancing several reproductive function-related characteristics. By administering putrescine, mating behavior and implantation in the PMOS-like hyperandrogenized mice were induced, which suggests that polyamine is a potential drug target for PMOS management.

This study conducted a cohort analysis to investigate the levels of polyamines in patients with PMOS and their correlation with various biochemical markers. The findings revealed that biochemical parameters, including BMI, PP-BS, PP-insulin levels, and testosterone concentrations, were significantly elevated in PMOS patients compared to healthy controls, corroborating previous research [38]. Furthermore, it was noted that circulating polyamines—namely, putrescine, spermidine, and spermine—were significantly reduced in PMOS patients relative to healthy subjects. This reduction is particularly significant given polyamines’ emerging role as crucial metabolic protectors. Specifically, the depletion of spermidine and spermine may hinder cellular autophagy and antioxidant defense mechanisms, potentially worsening insulin resistance and chronic inflammation—both of which are central to the pathogenesis of PMOS. From a therapeutic standpoint, these results imply that patients with PMOS could benefit from strategies aimed at restoring polyamine levels. Previous studies have shown that spermidine supplementation can mimic the effects of caloric restriction, thereby improving glucose metabolism and cardiovascular health. Therefore, exploring the role of polyamine-rich diets or targeted supplementation as adjunct therapies in PMOS presents a promising new avenue for clinical research [39]. To enhance understanding of these dynamics, the study employed DHEA-induced PMOS-like hyperandrogenized mouse models, which exhibit three common characteristics of PMOS: elevated body weight, blood glucose levels, and androgen levels. These symptoms are often observed in individuals with PMOS and replicated in animal models used to study the condition [40, 41]. Earlier reports have shown that this model is widely used for PMOS management [42, 43].

Since the thickening of the endometrium is one of the key features observed in women with PMOS [44]. The authors have also assessed changes in the thickness of the endometrium, as well as other cell layers of the mouse uterus, across different animal groups. The study revealed that DHEA-treated PMOS-like hyperandrogenized mice exhibited a significant increase in endometrial thickness, accompanied by a decrease in the number of endometrial glands, compared to control mice. Simultaneously, treatment with polyamines, PUT1, PUT2, and AGM could also improve the number of different follicle stages in the ovaries of PMOS-like hyperandrogenized mice.

However, among the polyamines, PUT1 treatment showed better improvement in ovarian and uterine morphology, as well as other parameters, such as body weight, compared to other polyamine doses. Henceforth, going by the data obtained from body weight, glucose level, and ovarian and uterine morphology, only one effective dose of polyamine, PUT1 (150 μg/250 g body weight), was selected to elicit the mechanism of how putrescine ameliorates PMOS condition in the PMOS-like hyperandrogenic mice model.

### The putrescine-GnRH axis: Implications for pubertal maturation

Earlier studies have shown that there is an increase in GnRH pulsatility in PMOS cases, causing a subsequent increase in ovarian androgen production thereby suggesting that novel ways to target the abnormal GnRH pulsatility might lead to PMOS management [45, 46]. Reports also suggest polyamines, particularly putrescine, can regulate hypothalamic GnRH expression and level in adult rodents [19] . It was also shown that putrescine could rescue hypothalamic GnRH expression in GT1-7 neuronal cells, even in the presence of hallmarks of aging parameters such as lactate and TNFα [47]. Another recent study has shown that polyamine supplementation can restore fertility in senescent mice [48]. In the present study, PUT1 treatment significantly lowered serum GnRH levels in PMOS-like hyperandrogenic mouse models; a similar trend was also observed in hypothalamic GnRH expression. However, PUT1 treatment also upregulated GnIH expression in the hypothalamus of PMOS mice. Thus, the present study may further strengthen the hypothesis that regulating abnormal GnRH pulsatility through polyamine supplementation can help manage PMOS conditions.

A study conducted by Fernandes et al. demonstrated that the administration of exogenous polyamines significantly enhances the hypothalamic expression of ODC1 in adult cyclic rats [19]. This finding suggests that exogenous polyamine treatment may boost endogenous polyamine anabolism in the hypothalamus, potentially influencing the regulation of hypothalamic GnRH expression and secretion. In our research, we similarly observed that putrescine (PUT1) treatment led to a marked increase in hypothalamic ODC1 and SMS expression in mice with PMOS-like hyperandrogenization. Conversely, we found a significant downregulation of ODC1, SMS, and SRM expression in the ovaries of these hyperandrogenized mice, which was similarly found in the blood of the PMOS patient. Several studies report that Az and Azin are key regulators of endogenous polyamine biosynthesis [49]. Notably, the exogenous administration of putrescine was effective in elevating the endogenous expression levels of these key markers associated with polyamine anabolism in the PMOS-like hyperandrogenized mouse ovary.

### Molecular pathways in the ovary and uterus

Studies indicated that women with PMOS exhibit elevated cytokines TNFα, IL6, and LEPTIN expression in their ovaries, and reducing these markers is a prerequisite for PMOS management [50]. Earlier studies have also shown that metformin successfully reduces inflammatory and metabolic markers, contributing to its overall therapeutic benefits in PMOS [51, 52]. The present study found that treatment with putrescine significantly reduced the expression of inflammatory markers, including Tnfα, Il6, and Leptin, in PMOS-like hyperandrogenized mice, further supporting the efficacy of putrescine in managing PMOS. Moreover, PUT1 also down-regulated Inha, a key regulator of premature ovarian failure [53], which is known to be up-regulated in women with PMOS [54]. The expression of the Akt gene was also upregulated in PMOS-like hyperandrogenized mice after PUT1 treatment, following a similar trend observed in earlier studies after melatonin or liraglutide treatment in PMOS mouse models, which ameliorates the PMOS conditions [42, 55] . Immunohistochemistry of ovaries from different groups of mice showed that the expression of FSHR in ovarian granulosa cells and corpus luteum of PMOS-like hyperandrogenized mice increased significantly after PUT1 treatment, whereas it was decreased in untreated hyperandrogenized mice. Studies have shown that decreased FSHR levels in ovarian granulosa cells in PMOS are due to higher androgen levels [56]. The level of the aromatase enzyme, essential for the production of estrogen, in the ovaries also followed a similar trend to FSHR. In contrast, the adiponectin expression in both the PMOS and the PUT1-treated PMOS group was lower than in the control. Adiponectin, a cytokine predominantly secreted by adipose tissue, plays a crucial role in metabolic processes and is closely linked to insulin sensitivity and inflammation. Research indicates that adiponectin expression in the ovaries of individuals with PMOS is markedly reduced, suggesting a potential dysregulation of this metabolic mediator in the context of PMOS pathophysiology [57].

Molecular markers of early pregnancy have already been reported to be decreased in expression in the PMOS-like hyperandrogenized mouse model. Several fundamental molecular interactions occur between the uterus and embryo during implantation; the involvement of feto-maternal interactions, matrix metalloproteinases, decidualization events, and the balance of sex hormones are central. Factors such as PGR, ESR1, ESR2, and integrin markers (ITGA4, ITGA5, ITGB1, and ITGB3) play a crucial role in the interaction between the uterine endometrium and the embryo during implantation. The uterine expression of progesterone, estrogen, and integrin markers are also known to be down-regulated in PMOS-like mouse models compared to control mice [33]. PUT1 treatment significantly elevated the expressions of PGR, ESR1, and ESR2, as well as the integrin markers, such as ITGA4, ITGA5, ITGB1, and ITGB3, in the uterus of non-mated PMOS-like hyperandrogenized mice compared to the untreated PMOS mouse model, which suggests improved uterine physiology and makes it more conducive for receiving an embryo. The attachment of blastocysts to the endometrial layer in the uterus triggers a decidualization event. It is well known that homeobox transcription factors, like HOX10 and HOX11, regulate decidualization events [58]. Moreover, the expressions of these homeobox genes are known to be down-regulated in PMOS-like mouse models compared to control mice [33]. Our observation revealed that PUT1 treatment efficiently elevated the expression of HOX10 and HOX11 in PMOS-like hyperandrogenized mice compared to untreated PMOS-like mice. During early pregnancy, tissue remodeling is required to provide proper implantation or attachment with endometrial stromal cells; this event is regulated by matrix MMPs and their inhibitors (TIMPs) [59]. Earlier studies suggest that an imbalance of MMPs and TIMPs leads to the termination of pregnancy [60]. Dhadal and Nampoothiri also showed that the expression of MMPs and TIMPs are downregulated in PMOS-like hyperandrogenized mice compared to the control [33]. Interestingly, PUT1 caused the elevation of the expression of uterine MMP2, MMP9, TIMP1, and TIMP3 in PMOS-like hyperandrogenized mice compared to the untreated ones. Another important factor responsible for a successful pregnancy is the expression of LIF and receptor genes. LIF is known to enhance decidualization in human endometrial stromal cells via the STAT3 phosphorylation following estrogen and progesterone treatment [61]. While assessing the levels of LIF, LIFR, and STAT3 after PUT1 treatment on PMOS-like hyperandrogenized mice, a significant increase in the levels of these markers was observed, suggesting an overall improvement in uterine physiology.

PMOS conditions are known to lower receptivity and lead to implantation failure in human and mouse models [62, 63], and the decline in pregnancy frequency is also attributed to insulin resistance, gestational diabetes, preeclampsia, and premature birth [64]. Therefore, the final aim of this study was to observe whether PUT1 can facilitate successful pregnancy in PMOS-like hyperandrogenized mice, as there is evidence stating that deprivation of putrescine caused failure in implantation [65]. Consistent with the previously stated hypothesis, treatment with putrescine (PUT1) demonstrated the ability to promote successful pregnancies in a mouse model exhibiting PMOS-like hyperandrogenism. This further supports the assertion that putrescine may effectively restore fertility in conditions resembling PMOS, positioning it as a potentially effective therapeutic strategy for managing PMOS.

### Strengths and limitations

To conclude, this study demonstrated that circulatory polyamine levels are lower in PMOS patients than in healthy controls. PUT1 treatment improved the reproductive outcomes of PMOS-like hyperandrogenized mice and can be a future therapeutic agent for PMOS management. Unlike untreated PMOS-like mice, mice treated with PUT1 were able to mate and experience normal implantation, along with increased markers of normal uterine physiology and implantation. Treatment with PUT1 in these PMOS-like hyperandrogenized mice not only improved GnRH and steroid levels, ovarian and uterine morphology, and reduced expression of inflammatory genes, but also increased fertility, as evidenced by implantation in the treated mice.

The authors recognize that the limited sample size in the animal phase of the study (n = 4) constitutes a significant limitation. Despite the results demonstrating robust statistical significance and consistency across gene and protein expression analyses, subsequent research with larger cohorts may be necessary to detect more nuanced neuroendocrine fluctuations.

## Conclusion

In summary, the present study correlates polyamines and PMOS for the first time. The study showed that polyamine administration, particularly putrescine treatment, ameliorates PMOS in hyperandrogenized mice. Putrescine treatment primarily regulates hypothalamic GnRH levels, thereby improving other reproductive features. By administering putrescine, mating behavior and implantation in the PMOS hyperandrogenized mice were induced, suggesting that polyamine can be a potential drug target for PMOS management.

## Supplementary Material

Supplementary_Figure_1_ioag139

Supplementary_Table_1_ioag139

Supplementary_Table_2_ioag139

Supplementary_Table_3_ioag139

Supplementary_Table_4_ioag139

## Data Availability

The datasets used and/or analysed during the current study are available from the corresponding authors upon reasonable request.
