# Supplementary material for: Reduced circulating polyamines in polyendocrine metabolic ovarian syndrome patients and the impact of putrescine on ovarian function and fertility in a murine PMOS model
Source: Biol Reprod. 2026 Jul 2;115(3):813–31. doi: 10.1093/biolre/ioag139 (PMC13578486; doi:10.1093/biolre/ioag139)
Supplement: Supplementary file 1 [file supplementary_figure_1_ioag139.docx]

**Figure S1**

**A	B**

Control	PMOS	PMOS+PUT1

**C	D**

**E	F	G**

Control	PMOS	PMOS+PUT1

**Figure S1:** (A) Graphical representation of the measurements of body weights of mice groups consisting of control mice, DHEA-treated PMOS-like hyperandrogenized mice, and PMOS-like hyperandrogenized mice treated with DFMO, respectively, at an interval of 5 days. The left image shows the individual body weights of each mouse in each group (n = 4) at each 5-day interval. The right image represents the comparison between the mean body weight of all mice across all groups (n=4). The group PMOS+DFMO were treated with DFMO for 5 days after 25 days of DHEA treatment. The data obtained from days 1 to 30, in 5-day intervals for all mice (n=4) from each group, were utilised to determine statistical significance. Results were expressed as mean ± S.E.M. Two-way ANOVA with Tukey’s post hoc test was used to analyse body weight measurement data. ****p<0.0001, ns p>0.05. (B) The bar diagrammatic representation in the panel shows the blood glucose levels of the different groups of mice at day 30. All value represented as mean ± S.E.M; n=3; ****p<0.0001, **p<0.01. (C) The H&E-stained histological sections of representative ovaries from each group of mice. The upper panel consists of microscopic images at 10X magnification, and the lower panel represents the 40X magnification of the portion marked with a black rectangle of the respective images at the upper panel. Here, PF means primary follicle, SF means secondary follicle, CL means corpus luteum, CF means cystic follicle, and OC means oocyte. (D) Graphical representation showing different classes of follicles in the ovaries of control mice, untreated PMOS-like mice, and PMOS-like mice treated with PUT1, DFMO. (E) The H&E-stained histological sections of the representative uterus from each group of mice show changes in perimetrium (yellow arrow), myometrium (red arrow), and endometrium (black arrow). The upper panel represents microscopic images at 10X magnification, and the lower panel represents 40X magnification of the portions marked with black rectangles in the respective upper panel images. The scale bar at 10X magnification images represents 200 µm and 10 µm at 40X magnification. (F) Graphical representation showing the number of cysts present in the ovaries of different groups of mice at day 30. All values are represented as mean ±S.E.M; n=4; ****p<0.0001, ***p<0.001. (G) Graphical representation showing the changes in the thickness of the luminal epithelium of the uterus from different groups of mice. All values are represented as mean ±S.E.M; n=4; ****p<0.0001, ***p<0.001.
