# Supplementary material for: Reduced circulating polyamines in polyendocrine metabolic ovarian syndrome patients and the impact of putrescine on ovarian function and fertility in a murine PMOS model
Source: Biol Reprod. 2026 Jul 2;115(3):813–31. doi: 10.1093/biolre/ioag139 (PMC13578486; doi:10.1093/biolre/ioag139)
Supplement: Supplementary file 2 [file supplementary_table_2_ioag139.docx]

**Table No. S2a:** The table presents differences in biochemical parameters and polyamine levels in the blood of PMOS patients compared to healthy individuals. All values are represented as mean ±S.E.M; n=30; ****p<0.0001, ***p<0.001, **p<0.01, *p<0.05, ns= p>0.05

| Characteristics | Control (n=30) | PMOS (n=30) | p value |
| --- | --- | --- | --- |
| Age (Years, mean, ±SD) | 26.47±6.5966 | 25.67±6.0018 | p<0.6309 |
| BMI (Kg/m^2^) | 21.87±0.73 | 25.64±0.85 | p<0.0015 |
| PP-insulin (µU/mL) | 37.38±7.31 | 85.03±21.03 | p<0.0366 |
| PPBS (mg/dL) | 82.52±3.37 | 97.63±5.19 | p<0.0181 |
| Testosterone (ng/dL) | 18.69±3.10 | 29.31±3.16 | p<0.0198 |
| Estradiol (pg/mL) | 101.0±64.48 | 95.24±60.31 | p<0.7216 |
| Putrescine (µM/100µl blood) | 0.339306 ±0.005 | 0.2788807±0.003 | p<0.0001 |
| Spermidine (µM/100µl blood) | 2.561051±0.04 | 2.356716±0.06 | p<0.0080 |
| Spermine (µM/100µl blood) | 1.934081219±0.05 | 1.593660967±0.05 | p<0.0001 |

**Table No. S2b:** The table represents individual biochemical parameters and polyamine levels in the blood of control population.

| Reg. No | Age | Oligo/  Amenorrhoea | Hyper Androgenism | Polycystic Ovaries | BMI (Kg/m^2^) | PPBS (mg/dL) | PP-insulin (µU/mL) | Testosterone (ng/dL) | Estradiol  (pg/mL) | Putrescine (µM/200µL Blood) | Spermidine (µM/200µL Blood) | Spermine (µM/200µL Blood) |
| --- | --- | --- | --- | --- | --- | --- | --- | --- | --- | --- | --- | --- |
| C1 | 18 | NO | NO | NO | 18.0 | 70 | 7.27 | 41.31 | 82.83 | 0.328582057 | 2.220236 | 2.213056047 |
| C2 | 19 | NO | NO | NO | 29.5 | 89 | 8.75 | 43.1 | 185.57 | 0.319501094 | 2.259339 | 1.957067847 |
| C3 | 19 | NO | NO | NO | 19.6 | 82 | 17.16 | 2.5 | 112.98 | 0.328544858 | 2.255772 | 1.58219469 |
| C4 | 19 | NO | YES | NO | 22.2 | 92 | 9.42 | 26.67 | 50.94 | 0.342545952 | 2.392669 | 1.815215339 |
| C5 | 20 | NO | YES | NO | 26.2 | 143 | 32.00 | 29.53 | 112.04 | 0.309870897 | 2.570441 | 1.779150442 |
| C6 | 20 | NO | NO | NO | 22.2 | 76 | 29.03 | 2.5 | 113.46 | 0.315308534 | 2.664685 | 1.681097345 |
| C7 | 20 | NO | NO | NO | 25.5 | 77 | 7.07 | 32.45 | 134.98 | 0.347181619 | 2.644031 | 1.912123894 |
| C8 | 21 | NO | NO | NO | 19.8 | 85 | 20.61 | 10.22 | 149.08 | 0.395273523 | 2.26085 | 1.679439528 |
| C9 | 21 | NO | NO | NO | 14.7 | 66 | 150.90 | 18.08 | 40.03 | 0.317495624 | 2.427323 | 1.865327434 |
| C10 | 22 | NO | YES | NO | 18.8 | 107 | 99.10 | 10.36 | 147.09 | 0.331280088 | 2.238654 | 1.662365782 |
| C11 | 23 | NO | NO | NO | 22.2 | 73 | 42.17 | 5.52 | 28.83 | 0.309911379 | 2.614079 | 1.463486726 |
| C12 | 23 | NO | NO | NO | 17.6 | 68 | 25.55 | 7.41 | 157 | 0.329212254 | 2.744921 | 2.420176991 |
| C13 | 23 | NO | NO | NO | 19.5 | 81 | 55.77 | 10.65 | 48.45 | 0.349793217 | 2.474079 | 2.168330383 |
| C14 | 23 | NO | NO | NO | 22.2 | 84 | 15.87 | 13.7 | 54.5 | 0.365393873 | 2.267937 | 1.609817109 |
| C15 | 24 | NO | NO | NO | 26.5 | 97 | 24.82 | 16.19 | 23.87 | 0.313952954 | 2.311976 | 1.66360472 |
| C16 | 25 | NO | NO | NO | 16.5 | 64 | 15.57 | 24.76 | 50.75 | 0.314243982 | 2.545858 | 1.744884956 |
| C17 | 26 | NO | YES | NO | 24.5 | 66 | 23.00 | 31.2 | 68.22 | 0.301321663 | 2.837685 | 1.977433628 |
| C18 | 26 | NO | NO | NO | 18.5 | 101 | 41.00 | 13.93 | 37.11 | 0.304718818 | 2.880898 | 1.847215339 |
| C19 | 27 | NO | NO | NO | 23.6 | 79 | 17.17 | 72 | 44.2 | 0.398690372 | 2.899409 | 1.413775811 |
| C20 | 28 | NO | NO | NO | 19.5 | 78 | 6.53 | 2.5 | 150.79 | 0.394601751 | 2.67326 | 2.322117994 |
| C21 | 30 | NO | YES | NO | 22.2 | 83 | 37.00 | 17.82 | 179.08 | 0.397589716 | 2.433724 | 2.25759292 |
| C22 | 31 | NO | NO | NO | 23.5 | 96 | 19.89 | 12.44 | 63.75 | 0.337708972 | 2.42585 | 2.486696165 |
| C23 | 31 | YES | NO | NO | 23.0 | 85 | 60.77 | 10.96 | 18.6 | 0.338145514 | 2.408197 | 2.128312684 |
| C24 | 32 | NO | NO | NO | 19.5 | 66 | 54.63 | 20.93 | 177.63 | 0.354239606 | 2.560929 | 1.899899705 |
| C25 | 34 | YES | NO | NO | 23.2 | 76 | 9.16 | 17.56 | 304.62 | 0.369115974 | 2.674213 | 1.803386431 |
| C26 | 35 | NO | NO | NO | 29.5 | 95 | 20.50 | 55.97 | 116.58 | 0.322434354 | 2.880787 | 2.572442478 |
| C27 | 36 | NO | NO | NO | 21.3 | 94 | 64.79 | 2.5 | 139.68 | 0.368442013 | 2.41278 | 1.606377581 |
| C28 | 39 | NO | NO | NO | 27.3 | 72 | 30.00 | 2.92 | 126.58 | 0.322983589 | 3.130795 | 2.028371681 |
| C29 | 39 | NO | NO | NO | 20.5 | 59 | 3.67 | 2.5 | 53.98 | 0.340455142 | 2.887512 | 2.001893805 |
| C30 | 40 | NO | NO | NO | 19.0 | 71 | 172.30 | 2.5 | 55.97 | 0.310631291 | 2.83263 | 2.040383481 |

**Table No. S2c:** The table represents individual biochemical parameters and polyamine levels in the blood of PMOS population.

| Reg. No | Age | Oligo/  Amenorrhoea | Hyper  Androgenism | Polycystic  Ovaries | BMI (Kg/m^2^) | PPBS (mg/dL) | PP-insulin (µU/mL) | Testosterone (ng/dL) | Estradiol  (pg/mL) | Putrescine (µM/200µL Blood) | Spermidine (µM/200µL Blood) | Spermine (µM/200µL Blood) |
| --- | --- | --- | --- | --- | --- | --- | --- | --- | --- | --- | --- | --- |
| P1 | 18 | YES | YES | YES | 19.6 | 71 | 42.00 | 18 | 34.22 | 0.242009 | 2.500126 | 1.514867 |
| P2 | 18 | YES | YES | YES | 27.6 | 106 | 558.00 | 16.9 | 219.8 | 0.273162 | 1.68385 | 1.332018 |
| P3 | 18 | YES | NO | YES | 18.7 | 72 | 36.45 | 19 | 110 | 0.276271 | 2.764709 | 1.359923 |
| P4 | 19 | YES | YES | YES | 27.7 | 95 | 116.50 | 5.14 | 81.22 | 0.280602 | 2.563402 | 1.43033 |
| P5 | 19 | YES | YES | YES | 22.3 | 101 | 34.77 | 21.63 | 70.91 | 0.30901 | 2.563402 | 1.439835 |
| P6 | 21 | YES | YES | YES | 31.0 | 123 | 33.00 | 45.83 | 49.07 | 0.270311 | 2.595528 | 1.50574 |
| P7 | 21 | YES | YES | YES | 25.8 | 92 | 4.56 | 39.82 | 70.91 | 0.302525 | 1.68385 | 1.31872 |
| P8 | 22 | YES | YES | YES | 24.0 | 110 | 206.00 | 35 | 78.67 | 0.259873 | 2.395472 | 1.91131 |
| P9 | 22 | YES | YES | YES | 21.2 | 78 | 89.00 | 33.91 | 60.62 | 0.268857 | 2.410669 | 1.169841 |
| P10 | 22 | YES | YES | YES | 22.8 | 145 | 9.32 | 27.48 | 59.21 | 0.271475 | 2.423394 | 1.695746 |
| P11 | 22 | YES | YES | YES | 17.6 | 56 | 5.82 | 22.67 | 301.21 | 0.27785 | 2.476307 | 1.775463 |
| P12 | 23 | NO | YES | YES | 31.6 | 117 | 39.13 | 30 | 54.11 | 0.24615 | 2.49937 | 1.983906 |
| P13 | 23 | YES | YES | YES | 24.9 | 140 | 77.00 | 32.34 | 34.17 | 0.250534 | 2.451961 | 1.678077 |
| P14 | 23 | YES | NO | YES | 28.5 | 95 | 22.50 | 21.65 | 92.9 | 0.305523 | 2.764709 | 1.01974 |
| P15 | 24 | YES | YES | YES | 27.5 | 125 | 94.26 | 34.24 | 63.52 | 0.28586 | 2.199094 | 1.92813 |
| P16 | 24 | YES | YES | YES | 24.5 | 85 | 94.26 | 34.24 | 63.52 | 0.296295 | 2.738772 | 1.529882 |
| P17 | 26 | NO | YES | YES | 28.8 | 93 | 9.88 | 20.15 | 153.46 | 0.303973 | 2.294283 | 1.77964 |
| P18 | 26 | YES | YES | YES | 34.7 | 107 | 10.00 | 2.5 | 63.67 | 0.270278 | 2.738772 | 1.512484 |
| P19 | 26 | YES | YES | YES | 22.5 | 101 | 101.50 | 19.48 | 76.01 | 0.282536 | 1.853953 | 1.928159 |
| P20 | 26 | YES | YES | YES | 26.7 | 164 | 134.00 | 26.34 | 69.95 | 0.257783 | 2.010551 | 1.911558 |
| P21 | 27 | NO | YES | YES | 22.9 | 87 | 17.40 | 10.88 | 178 | 0.232047 | 2.134449 | 1.498065 |
| P22 | 27 | YES | YES | YES | 24.5 | 86 | 22.14 | 38.11 | 159.29 | 0.291478 | 2.500961 | 1.361717 |
| P23 | 27 | YES | YES | YES | 26.2 | 97 | 48.99 | 13.99 | 156.35 | 0.305536 | 2.476307 | 1.533457 |
| P24 | 31 | NO | YES | YES | 19.8 | 77 | 45.25 | 65.78 | 71.55 | 0.299325 | 2.595528 | 1.66282 |
| P25 | 33 | YES | YES | YES | 30.2 | 101 | 46.11 | 83.4 | 104.53 | 0.266869 | 1.819551 | 1.940372 |
| P26 | 34 | YES | YES | YES | 33.0 | 61 | 366.00 | 58.98 | 40.69 | 0.273914 | 2.294283 | 1.923292 |
| P27 | 34 | YES | YES | YES | 27.2 | 65 | 111.00 | 38.96 | 145.28 | 0.294108 | 2.347815 | 1.542903 |
| P28 | 36 | NO | YES | YES | 21.8 | 124 | 90.05 | 20.43 | 47 | 0.280907 | 1.772528 | 1.528814 |
| P29 | 38 | YES | YES | YES | 26.7 | 70 | 16.67 | 19.45 | 88.03 | 0.291582 | 2.724496 | 1.089463 |
| P30 | 40 | YES | YES | YES | 29.0 | 85 | 69.45 | 22.58 | 59.21 | 0.299779 | 2.423394 | 1.782389 |
