# Supplementary material for: Reduced circulating polyamines in polyendocrine metabolic ovarian syndrome patients and the impact of putrescine on ovarian function and fertility in a murine PMOS model
Source: Biol Reprod. 2026 Jul 2;115(3):813–31. doi: 10.1093/biolre/ioag139 (PMC13578486; doi:10.1093/biolre/ioag139)
Supplement: Supplementary file 3 [file supplementary_table_1_ioag139.doc]

**Table S1:** List of primers used in qRT-PCR studies.

| **Primer** | **Accession No.** | **Sequence of the primer (5’🡪3’)** | **Annealing temperature** | **Amplicon length (bp)** |
| --- | --- | --- | --- | --- |
| ODC1 | NM_001409644.1 | FP: CCAAGCCAGACGAGAAGTATT  RP: CATGCATTTCAGGCAGGTTAC | 60°C | 99 |
| SMS | NM_009214.4 | FP: GATGTGCAAGGCAAACAAGAA  RP: CGCGAACTATGGGTGGTAAT | 60°C | 115 |
| GnRH | NM_008145.4 | FP: TTTCACATCCAAACAGAGTGGAC  RP: GTCACAAGCCTCAGGGTCAA | 60°C | 357 |
| GnIH | NM_021892.2 | FP: CTGCCCCTGAGATTTGGAAG  RP: CAGGGGTTTCTGTGGCAAAT | 60°C | 165 |
| INHA | NM_010564.5 | FP: TTTTCCCAGCTACAGGTGCC  RP: GTTGGGATGGCCGGAATACA | 60°C | 105 |
| IL6RA | NM_010559.3 | FP: TCATCCATGATGCCTTGCGA  RP: TCCTGGGCTCTGCTATCCAA | 60°C | 129 |
| AKT | NM_009652.4 | FP: AAGGACCCTACACAGAGGCT  RP: AAGGTGGGCTCAGCTTCTTC | 60°C | 118 |
| TNFα | NM_013693.3 | FP: TAGCCCACGTCGTAGCAAAC  RP: ACAAGGTACAACCCATCGGC | 60°C | 136 |
| LEPTIN | NM_008493.3 | FP: ACATTTCACACACGCAGTCG  RP: ACTCAGAATGGGGTGAAGCC | 60°C | 83 |
| β-actin | NM_007393.5 | FP: TTACTGCTCTGGCTCCTAGC  RP: CAGCTCAGTAACAGTCCGC | 60°C | 198 |
| ESR1 | NM_007956.5 | FP: TTCTTCTCAAGCAGGTGGCCC  RP: GCGAGTTACAGACTGGCTCC | 60°C | 70 |
| ESR2 | NM_207707.1 | FP: CCTCGTTCTGGACAGGTCCTC  RP: CCTTGGGACAGCACTCTTCG | 60°C | 70 |
| HOX10 | NM_001301427.1 | FP: GCAGCGTCATGGCTGAGTAT  RP: AAGCCATCTTGAACCAGTAGC | 60°C | 136 |
| PGR | NM_008829.2 | FP: CATGGTCCTTGGAGGTCGTA  RP: CTCTCGTTAGGAAGGCCCAC | 60°C | 105 |
| ITGA4 | NM_010576.4 | FP: AGGCAGAGTCTCCGTCAAGA  RP: GGGGTAAGGATGTCTCGCAC | 60°C | 145 |
| ITGB3 | NM_016780.2 | FP: CTCCCCTCCGCAGGAAAAG  RP: TGGTACAGATGTTGGACTCTCC | 60°C | 171 |
| MMP2 | NM_008610.3 | FP: AACGGTCGGGAATACAGCAG  RP: CCATGGTAAACAAGGCTTCATGG | 60°C | 130 |
| MMP9 | NM_013599.5 | FP: TGGTCTTCCCCAAAGACCTG  RP: AGCGGTACAAGTATGCCTCTG | 60°C | 72 |
| TIMP1 | NM_001044384.1 | FP: GCAACTCGGACCTGGTCATAA  RP: CGCTGGTATAAGGTGGTCTCG | 60°C | 73 |
| TIMP3 | NM_011595.2 | FP: GGCTTCAGTAAGATGCCCCA  RP: CTTCATACACGCGCCCTGTC | 60°C | 118 |
| HOX11 | XM_006526915.4 | FP: CCTGGATGGAGAGTAACCGC  RP: GGCGTGTTACAGTGAAGGGT | 60°C | 75 |
| LIF | NM_008501.3 | FP: ACAACTGTGCAGACTGTGGA  RP: CAGTAAGGGCACAATCCCTGC | 60°C | 174 |
| LIFR | BC031929.1 | FP: TGGGTGCACTAACCAGGGAT  RP: AGTGACCGCTCAAACAGACT | 60°C | 140 |
| STAT3 | NM_213659.3 | FP: GCAATACCATTGACCTGCCG  RP: ACGTGAGCGACTCAAACTGC | 60°C | 114 |
| ITGA5 | NM_008402.4 | FP: GGTCGCCTATCTTCGGGATG  RP: TGAACTGGTTCAGGATGGGC | 60°C | 131 |
| AZ | NM_008753.4 | FP: CGCACCATGCCGCTTCTTA  RP: ATCCCGCTGACTGTTCCCT | 60°C | 169 |
| AZIN | XM_006520114.5 | PF: ATTGACGATGCGAACTACTCCG  RP: TTCCCAAGATCCCCCACAAAA | 60°C | 128 |
