# Supplementary material for: Reduced circulating polyamines in polyendocrine metabolic ovarian syndrome patients and the impact of putrescine on ovarian function and fertility in a murine PMOS model
Source: Biol Reprod. 2026 Jul 2;115(3):813–31. doi: 10.1093/biolre/ioag139 (PMC13578486; doi:10.1093/biolre/ioag139)
Supplement: Supplementary file 4 [file supplementary_table_3_ioag139.doc]

**Table S3:** Tissue-specific localisation of ODC1, SMS, and SRM was examined in the ovaries of control mice, as well as in mice exhibiting PMOS-like hyperandrogenization and those treated with PUT1. Quantitative data are presented as the presence (+) or absence (-) of markers.

| Protein | Characteristics | Control | PMOS | PMOS+PUT1 |
| --- | --- | --- | --- | --- |
| ODC1 | Primordial follicles | +++ | + | ++ |
|  | Primary & secondary follicles | +++ | + | ++ |
|  | Graafian follicles | +++ | - | ++ |
|  | Corpus luteum | ++ | - | + |
|  | Granulosa cells | +++ | - | ++ |
|  | Theca cells | +++ | - | ++ |
|  | Oocytes | +++ | - | ++ |
|  | Cystic follicles | - | ++ | - |
| SMS | Primordial follicles | +++ | - | + |
|  | Primary & secondary follicles | +++ | - | ++ |
|  | Graafian follicles | +++ | - | ++ |
|  | Corpus luteum | +++ | - | ++ |
|  | Granulosa cells | ++ | - | ++ |
|  | Theca cells | +++ | - | + |
|  | Oocytes | ++ | - | + |
|  | Cystic follicles | - | ++ | - |
| SRM | Primordial follicles | ++ | - | + |
|  | Primary & secondary follicles | +++ | - | ++ |
|  | Graafian follicles | ++ | - | + |
|  | Corpus luteum | ++ | - | + |
|  | Granulosa cells | ++ | - | + |
|  | Theca cells | +++ | - | + |
|  | Oocytes | ++ | - | + |
|  | Cystic follicles | - | + | - |
