# Supplementary material for: Reduced circulating polyamines in polyendocrine metabolic ovarian syndrome patients and the impact of putrescine on ovarian function and fertility in a murine PMOS model
Source: Biol Reprod. 2026 Jul 2;115(3):813–31. doi: 10.1093/biolre/ioag139 (PMC13578486; doi:10.1093/biolre/ioag139)
Supplement: Supplementary file 5 [file supplementary_table_4_ioag139.doc]

**Table S4:** Tissue-specific localisation of ESRα, ESRβ, and PGR was examined in the uterus of control mice, as well as in mice exhibiting PMOS-like hyper androgenization and those treated with PUT1. Quantitative data are presented as the presence (+) or absence (-) of markers.

| Protein | Characteristics | Control | PMOS | PMOS+PUT1 |
| --- | --- | --- | --- | --- |
| ESRα | Luminal epithelium | +++ | + | +++ |
|  | Glandular epithelium | +++ | - | +++ |
|  | Stroma | ++ | - | ++ |
| ESRβ | Luminal epithelium | +++ | + | +++ |
|  | Glandular epithelium | +++ | + | ++ |
|  | Stroma | ++ | - | + |
| PGR | Luminal epithelium | +++ | + | +++ |
|  | Glandular epithelium | +++ | - | +++ |
|  | Stroma | +++ | - | ++ |
